# The pro-oncogenic noncanonical activity of a RAS•GTP:RanGAP1 complex facilitates nuclear protein export

**DOI:** 10.1038/s43018-024-00847-5

**Published:** 2024-11-11

**Authors:** Brajendra K. Tripathi, Nicole H. Hirsh, Xiaolan Qian, Marian E. Durkin, Dunrui Wang, Alex G. Papageorge, Ross Lake, Yvonne A. Evrard, Adam I. Marcus, Suresh S. Ramalingam, Mary Dasso, Karen H. Vousden, James H. Doroshow, Kylie J. Walters, Douglas R. Lowy

**Affiliations:** 1 Laboratory of Cellular Oncology, Center for Cancer Research, National Cancer Institute, National Institutes of Health, Bethesda, MD USA; 2 Laboratory of Genitourinary Cancer Pathogenesis, Center for Cancer Research, National Cancer Institute, National Institutes of Health, Bethesda, MD USA; 3 Leidos Biomedical Research, Inc., Frederick National Laboratory for Cancer Research, Frederick, MD USA; 4 Winship Cancer Institute, Emory University, Atlanta, GA USA; 5 Division of Molecular and Cellular Biology, National Institute for Child Health and Human Development, Bethesda, MD USA; 6 p53 and Metabolism Laboratory, The Francis Crick Institute, London, UK; 7 Developmental Therapeutics Branch, Center for Cancer Research, National Cancer Institute, National Institutes of Health, Bethesda, MD USA; 8 Center for Structural Biology, Center for Cancer Research, National Cancer Institute, National Institutes of Health, Frederick, MD USA

**Keywords:** Non-small-cell lung cancer, Oncogenes, Tumour-suppressor proteins, Cancer

## Abstract

Canonical RAS signaling, including PI3K/AKT- and RAF/MEK-dependent activities, results mainly from RAS•GTP interaction with its effectors at the plasma membrane. Here, we identified a fundamental, oncogenic, noncanonical RAS•GTP activity that increases XPO1-dependent export of nuclear protein cargo into the cytoplasm and is independent of PI3K/AKT and RAF/MEK signaling. This RAS-dependent step acts downstream from XPO1 binding to nuclear protein cargo and is mediated by a perinuclear protein complex between RAS•GTP and RanGAP1 that facilitates hydrolysis of Ran•GTP to Ran•GDP, which promotes release of nuclear protein cargo into the cytoplasm. The export of nuclear EZH2, which promotes cytoplasmic degradation of the DLC1 tumor suppressor protein, is a biologically important component of this pro-oncogenic activity. Conversely, preventing nuclear protein export contributes to the antitumor activity of KRAS inhibition, which can be further augmented by reactivating the tumor suppressor activity of DLC1 or potentially combining RAS inhibitors with other cancer treatments.

## Abstract (web summary)

Tripathi et al. identify a noncanonical RAS•GTP activity that increases XPO1-dependent export of nuclear proteins into the cytoplasm. This depends on a RAS–RanGAP1 complex, and this export function contributes to RAS oncogenic activity.

## Main

Cancer arises as a multistep process that involves genetic, epigenetic and other nongenetic changes^1^. Modifications include alterations in the expression of genes, such as the Ran GTPase and its regulators or effectors, which together are critical for importing the vast majority of nuclear proteins and exporting a subset of them back into the cytoplasm^2,3^. However, the underlying mechanisms responsible for these changes and their pathogenetic role in cancer remain underexplored.

We have been studying the deleted in liver cancer 1 (*DLC1*) tumor suppressor gene, which encodes a cytoplasmic focal adhesion protein, and recently reported that in lung cancer, cytoplasmic EZH2 methylates a specific DLC1 lysine residue, causing DLC1 ubiquitination and subsequent proteasome-dependent degradation^4^. An alternate mechanism of ubiquitin-dependent proteasomal degradation of DLC1 was recently described in breast cancer^5^.

As the EZH2 lysine methyltransferase is usually considered a nuclear protein whose main activity is the methylation of Lys 27 on histone H3 (refs. ^6–8^), we investigated the origin of cytoplasmic EZH2. Our analysis of the nuclear protein export process has unexpectedly implicated KRAS, which is frequently mutated in pulmonary, pancreatic and colorectal cancer and in other tumor types^9^. Our findings indicate that inhibition of mutant KRAS in lung cancer can phenocopy inhibition of the nuclear export protein exportin 1 (XPO1)^10,11^, including the effects on EZH2 and DLC1, whereas increased KRAS activity has the opposite effects. Despite these similarities between the pharmacologic inhibition of XPO1 and KRAS, they inhibit protein export at different steps.

The vast majority of RAS oncogenic activity is thought to be mediated by its canonical signaling, with the best studied effectors being RAF and PI3K family members, which activate MEK/ERK and AKT/mTOR pathways, respectively^9^. However, we find that the effects of KRAS inhibition (KRASi) on XPO1-dependent activity are independent of MEK and PI3K signaling. Our investigation of the relationship between RAS and nuclear protein export has resulted in the surprising observation that GTP-bound RAS (RAS•GTP) forms a stable complex with Ran GTPase-activating protein 1 (RanGAP1), a major regulator of Ran•GTP. The complex facilitates hydrolysis of Ran•GTP to Ran•GDP and the release of cargo proteins exported by XPO1 into the cytoplasm. These findings implicate nuclear protein export as a critical noncanonical pro-oncogenic RAS function, highlight the role of this function in the antitumor activity of RAS inhibition and suggest possible drug combinations that may cooperate with RAS inhibition.

## Results

### Inhibition of XPO1 or KRAS reduces cytoplasmic EZH2 and increases DLC1

**Fig. 1:**
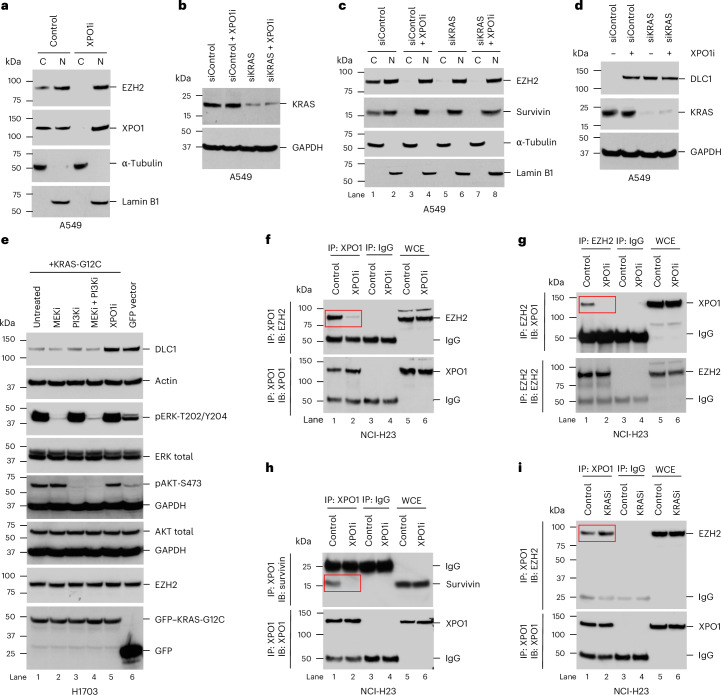
RAS regulates nuclear protein export independently of PI3K and MEK signaling. **a**, XPO1i (selinexor) prevented cytoplasmic export of EZH2 and XPO1. α-tubulin and lamin B1 were used as cytoplasmic and nuclear marker proteins, respectively; C, cytoplasmic; N, nuclear. **b**–**d**, siRNA knockdown of *KRAS* (**b**) or XPO1i by selinexor reduced cytoplasmic EZH2 (**c**) and increased DLC1 (**d**). Combined treatment with selinexor and *KRAS* siRNA did not further increase the response. **e**, Stable transfection of mutant KRAS-G12C in H1703 cells decreased DLC1 expression, which was not affected by MEKi (U0126-ethanol) or PI3Ki (wortmannin) but was increased by XPO1i (selinexor). Wortmannin inhibited PI3K activity (measured by pAKT-S473), and U0126-ethanol inhibited MEK activity (measured by pERK-T202/Y204) in all treated samples. **f**–**i**, In the KRAS-G12C NCI-H23 line, selinexor prevented complex formation between XPO1 and EZH2 (**f** and **g**) and between XPO1 and survivin (**h**), whereas complex formation was not prevented by the KRAS-G12C inhibitor sotorasib (**i**). Two independent experiments were performed for each image, with similar results; IB, immunoblot; IP, immunoprecipitation; WCE, whole-cell extract. Source data

**Extended Data Fig. 1:**
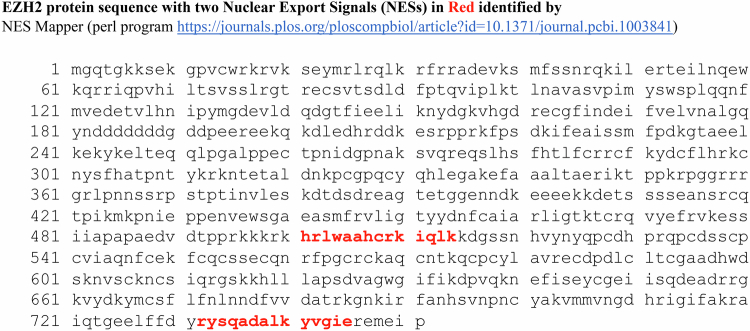
EZH2 protein sequence contains two consensus nuclear export signals, highlighted in red. EZH2 protein sequence with two Nuclear Export Signals (NES) in red identified by NES Mapper (per) program.

The previously observed increase in cytoplasmic EZH2 protein expression in lung cancer^4^ could result from incomplete EZH2 import into the nucleus, increased export of nuclear EZH2 into the cytoplasm or both processes. XPO1 is responsible for the cytoplasmic export of most nuclear proteins, and we identified two putative nuclear export signals in EZH2 protein similar to those present in known XPO1 cargo proteins (Extended Data Fig. 1), which suggested that EZH2 export might be mediated by XPO1. Therefore, we evaluated the impact of XPO1 inhibition (XPO1i) on the steady-state level of cytoplasmic EZH2 by using the XPO1-specific inhibitor selinexor in the A549 non-small cell lung cancer (NSCLC) cell line, which expresses *DLC1* mRNA but lacks readily detectable DLC1 protein^4^. Selinexor (XPO1i) treatment resulted in undetectable cytoplasmic EZH2 and XPO1, whereas EZH2 expression in the nucleus was not perturbed (Fig. 1a); α-tubulin and lamin B1 were used as cytoplasmic and nuclear marker proteins, respectively.

**Extended Data Fig. 2:**
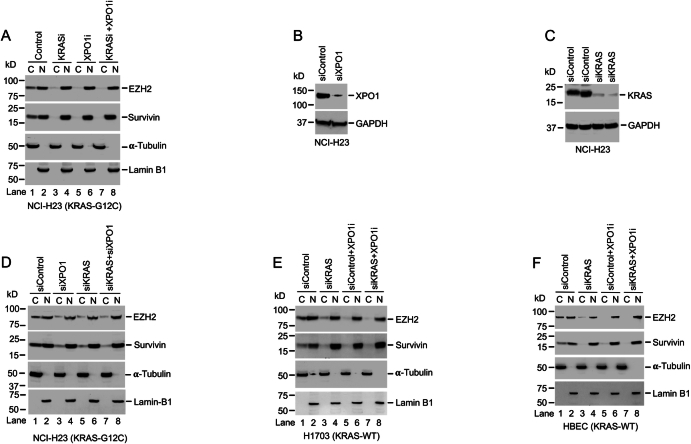
XPO1 inhibition or RAS inhibition prevents EZH2 and Survivin export from nucleus to cytoplasm. **a** In the NCI-H23 line, which harbors mutant KRAS-G12C, the XPO1 inhibitor selinexor or the KRAS-G12C inhibitor sotorasib prevented nuclear export of EZH2 and Survivin into the cytoplasm. α-tubulin and lamin B1 were used, respectively, as cytoplasmic and nuclear marker proteins. C, Cytoplasmic; N, Nuclear. **b-d** siRNA knockdown of XPO1 (**b**) or KRAS (**c**) prevented nuclear export of EZH2 and Survivin into the cytoplasm (**d**). GAPDH was used a loading control. **e,f** Results from XPO1 inhibition or KRAS knockdown in H1703 (**e**) and HBEC (**f**) lines were similar to those in NCI-H23 line (**a,d**). Two independent experiments were performed for each image, with similar results. Source data

Our recent publication presented preliminary evidence that KRAS could affect cytoplasmic EZH2 and DLC1 (ref. ^4^). To further explore this relationship, we knocked down *KRAS* expression by short interfering RNA (siRNA) in A549 cells (Fig. 1b), which harbor a G12S mutant (*KRAS*^G12S^) and compared the effect of *KRAS* knockdown to XPO1i (Fig. 1b–d). Survivin protein expression was also examined (Fig. 1c), as its nuclear export is known to be XPO1 dependent^12^. The results indicated that *KRAS* knockdown and XPO1i phenocopied each other with respect to the reduction of cytoplasmic EZH2 protein and cytoplasmic survivin protein expression (Fig. 1c, compare lanes 3 and 5) and similar increases in DLC1 protein expression (Fig. 1d). Combined inhibition of XPO1 and KRAS did not further increase DLC1 protein expression compared to each inhibition alone (Fig. 1d), suggesting that XPO1 and KRAS may act in the same pathway. An analogous reduction of cytoplasmic survivin protein and EZH2 protein expression was detected in the NCI-H23 NSCLC line, which carries mutant *KRAS*^G12C^, when the KRAS-G12C-specific inhibitor (KRASi) sotorasib or *KRAS* siRNA was used with XPO1i or *XPO1* siRNA (Extended Data Fig. 2a–d). Similar results were seen with *KRAS* siRNA and XPO1i in the H1703 NSCLC line, whose *KRAS* is wild type, and in the human bronchial epithelial cell (HBEC) line, which is immortalized but nontransformed^13^ (Extended Data Fig. 2e,f).

### KRASi of cytoplasmic export is independent of MAPK and PI3K signaling

**Extended Data Fig. 3:**
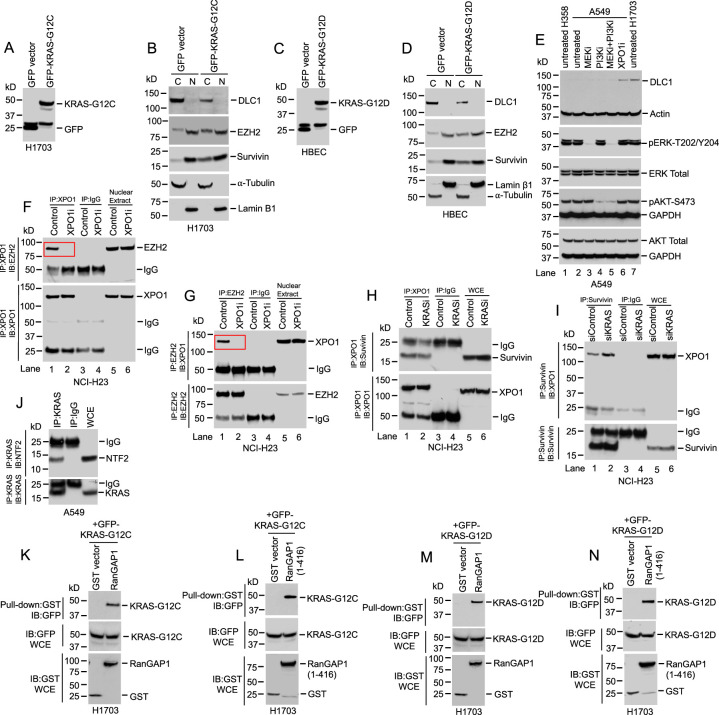
Overexpression of mutant KRAS increases nuclear export of EZH2 and Survivin, which is independent of PI3K and MEK signaling; KRAS inhibition does not prevent XPO1:Survivin complex formation. **a-d** Overexpression of KRAS-G12C in H1703 (**a,b**) or KRAS-G12D in HBEC (**c,d**) lines increased the level of cytoplasmic EZH2 and cytoplasmic Survivin and decreased the cytoplasmic DLC1 protein. α-tubulin and lamin B1 were used, respectively, as cytoplasmic and nuclear marker proteins. C, Cytoplasmic; N, Nuclear. **e** In A549 cells, MEK inhibitor U0126-ethanol and PI3K inhibitor wortmannin did not affect the level of DLC1 protein, while the XPO1 inhibitor selinexor did increase DLC1 protein. U0126-ethanol inhibited MEK activity (measured by pERK-T202/Y204) and wortmannin inhibited PI3K activity (measured by pAKT-S473) in all treated samples. **f,g** In the KRAS-G12C NCI-H23 line, selinexor prevented the complex formation between XPO1 and EZH2 in the nucleus, as it is confirmed from the purified nuclear extract. **h,i** KRAS inhibition by sotorasib (**h**) or siRNA knockdown of KRAS (**i**) did not prevent XPO1:Survivin complex formation. Lysates from NCI-H23 cells treated without or with sotorasib or KRAS siRNA were IP with antibodies to XPO1, Survivin, or mock IgG, followed by IB with antibodies to Survivin or XPO1. WCE, whole cell extract. **j** Complex formation between KRAS and NTF2 in A549 cells. Lysates from A549 cells were IP with antibodies to KRAS or mock IgG, followed by IB with antibodies to NTF2 or KRAS. **k-n** Overexpressed KRAS-G12C (**k,l**) or KRAS-G12D (**m,n**) formed a complex with full-length RanGAP1 (**k,m**) and the RanGAP1 catalytic domain (**l,n**). Two independent experiments were performed for each image, with similar results. Source data

Given that KRASi reduced cytoplasmic EZH2 protein expression and increased DLC1 protein expression, we tested whether overexpression of mutant *KRAS* produces the opposite phenotype. Indeed, transfection of a green fluorescent protein (GFP)-tagged *KRAS*^G12C^ construct into H1703 cells led to reduced endogenous DLC1 protein expression (Fig. 1e, compare lanes 1 and 6) and increased cytoplasmic EZH2 protein and cytoplasmic survivin protein expression (Extended Data Fig. 3a,b). Similar results were observed when GFP-tagged *KRAS*^G12D^ was transfected into the HBEC line (Extended Data Fig. 3c,d).

To examine if this phenomenon depends on canonical RAS signaling, we tested whether inhibition of the MEK/ERK and AKT/mTOR pathways affects the ability of mutant KRAS to increase cytoplasmic EZH2 protein expression and reduce DLC1 protein expression (Fig. 1e). Unexpectedly, when H1703 cells stably transfected with *KRAS*^G12C^ were treated with a MEK inhibitor (U0126-ethanol) or a PI3K inhibitor (wortmannin) singly or in combination (Fig. 1e, lanes 2–4), they did not affect EZH2 or DLC1 protein levels, although both inhibitors prevented phosphorylation of their known targets, pERK-T202/Y204 for MEK and pAKT-S473 for PI3K (Fig. 1e). By contrast, XPO1i increased DLC1 protein expression (Fig. 1e), as expected. Similarly, MEK inhibition (MEKi) and PI3K inhibition (PI3Ki; selumetinib and copanlisib, respectively) of the A549 line did not change the expression of DLC1 protein, unlike XPO1i (Extended Data Fig. 3e). From these results, we infer that the effects of KRAS on XPO1-dependent signaling are independent of canonical KRAS signaling.

### Inhibition of KRAS or XPO1 prevents XPO1-dependent protein export by distinct mechanisms

Despite the phenotypic similarities between XPO1i and KRASi, we speculated that they might act by different mechanisms. XPO1i prevents protein export by interfering with the interaction between XPO1 and its protein cargo substrates^11^, thereby preventing formation of the trimeric complex composed of XPO1 and its cargos, such as survivin and EZH2, together with Ran•GTP. By contrast, if KRASi were preventing nuclear export at a later step, formation of these complexes would not be prevented by KRASi. Therefore, we examined the interaction between XPO1 and EZH2 or survivin in the NCI-H23 line to compare the effects of XPO1i and KRASi. Although XPO1i prevented complex formation between XPO1 and EZH2 (Fig. 1f,g) or survivin (Fig. 1h) in the nucleus (Extended Data Fig. 3f,g), KRASi or siRNA knockdown of *KRAS* did not prevent complex formation (Fig. 1i and Extended Data Fig. 3h,i), supporting the conclusion that KRAS acts at a step after XPO1–cargo complex formation.

### KRAS•GTP forms an endogenous complex with RanGAP1

A PubMed search of previous publications that might have identified a complex between RAS and a molecule implicated in nucleocytoplasmic shuttling led us to an article by Wurzer et al., who reported a complex between overexpressed mutant HRAS protein and endogenous nuclear transport factor 2 (NTF2) protein^14^, which is implicated in nuclear protein import through its binding to Ran•GDP^15^. Although it was straightforward to identify an analogous endogenous complex between KRAS and NTF2 in A549 cells (Extended Data Fig. 3j), we did not identify a clear homology between NTF2 and other proteins known to interact with RAS.

**Fig. 2:**
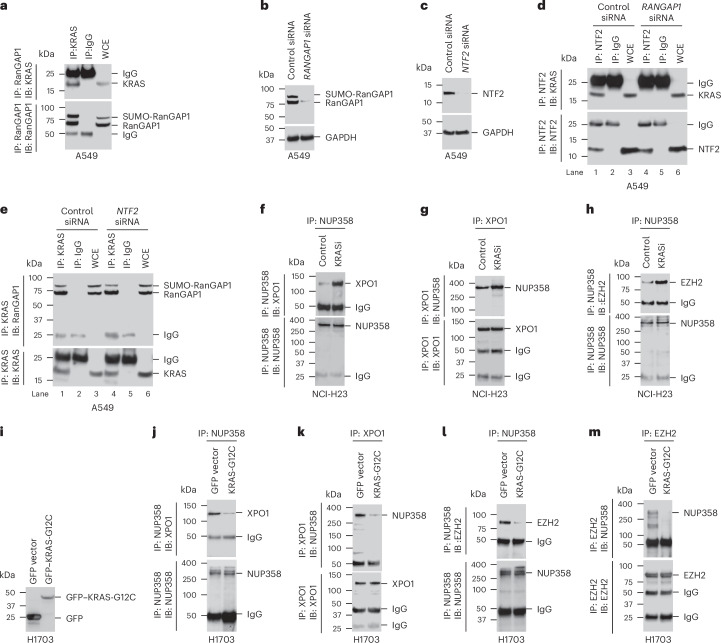
KRAS and RanGAP1 form a protein complex that regulates the release of nuclear protein cargo (EZH2) from the NPC. **a**, Complex formation between KRAS and RanGAP1 in A549 cells. Lysates from A549 cells were immunoprecipitated with antibody to RanGAP1 or mock IgG, followed by immunoblotting with antibody to KRAS or RanGAP1. **b**–**e**, siRNA knockdown of RanGAP1 (**b**) abolished the KRAS–NTF2 complex (**d**; lane 4), whereas siRNA knockdown of *NTF2* (**c**) did not affect the KRAS–RanGAP1 complex (**e**; lane 4). GAPDH was used as a loading control. **f**–**h**, Sotorasib treatment increased the complexes between NUP358 and XPO1 (**f** and **g**) and between NUP358 and EZH2 (**h**). Lysates from sotorasib-treated or sotorasib-untreated NCI-H23 cells were immunoprecipitated with antibody to NUP358 or XPO1 or mock IgG, followed by immunoblotting with antibody to NUP358, XPO1 or EZH2. **i**–**m**, Overexpressing mutant KRAS-G12C (**i**) decreased the complexes between NUP358 and XPO1 (**j** and **k**) and between NUP358 and EZH2 (**l** and **m**). Lysates from H1703 cells overexpressing mutant KRAS-G12C were immunoprecipitated with antibody to NUP358, XPO1 or EZH2 or mock IgG, followed by immunoblotting with antibody to NUP358, XPO1 or EZH2. Two independent experiments were performed for each image with similar results. Source data

We therefore speculated that KRAS might be part of a complex that includes a different protein involved with Ran regulation^15,16^ and might have homology with other known RAS binding proteins and, possibly, linkage to XPO1-dependent function. One such protein complex includes NUP358 (also known as RanBP2), which is located on the cytoplasmic face of the nuclear pore complex (NPC); NUP358 can bind both Ran•GTP and Ran•GDP as well as other proteins that interact with Ran^17,18^. We therefore considered a possible direct interaction between KRAS and RanGAP1 and that hydrolysis of Ran•GTP to Ran•GDP when bound to NUP358 leads to the cytoplasmic release of the nuclear export cargo from XPO1, the last step in the XPO1-dependent nuclear export process^19^. To evaluate this possibility, we first tested and confirmed that KRAS forms a complex with RanGAP1 in A549 cells (Fig. 2a) and then determined, with siRNA knockdown of RanGAP1 or NFT2 (Fig. 2b,c), that the KRAS–NFT2 complex required RanGAP1, whereas the KRAS–RanGAP1 complex did not require NTF2 (Fig. 2d,e, lanes 1 versus 4). The RanGAP1 doublet in Fig. 2a,b,e is attributable to a slower migrating form that is SUMOylated and a faster migrating form that is not^20^.

**Fig. 3:**
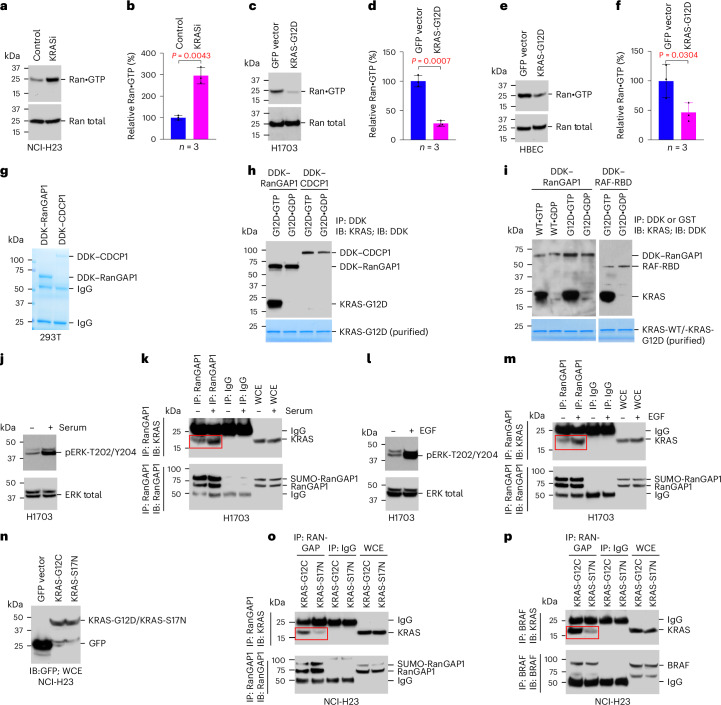
RAS•GTP and RanGAP1 interact directly and regulate the level of cytoplasmic Ran•GTP. **a**–**f**, KRASi by treatment with sotorasib increased cytoplasmic Ran•GTP in NCI-H23 cells (**a** and **b**), whereas overexpression of KRAS-G12D decreased cytoplasmic Ran•GTP in H1703 (**c** and **d**) and HBEC (**e** and **f**) cell lines. In **b**, **d** and **f**, bar graphs represent mean values of Ran•GTP, and error bars represent s.d.; *n* = 3 independent experiments. For the statistical analyses for **b**, **d** and **f**, a parametric unpaired one-tailed *t*-test with Welch’s correction was performed using Prism software, and no adjustments were made for multiple comparisons; *P* = 0.0043 for **b**, *P* = 0.0007 for **d**, and *P* = 0.0304 for **f**. **g**–**i**, Purified RanGAP1 (**g**) bound to KRAS-G12D•GTP but not to KRAS-G12D•GDP (**h**). Purified CDCP1 (**g**) was used as a negative control (**h**). Purified RanGAP1 binds to the GTP-bound form of wild-type KRAS (KRAS-WT) and KRAS-G12D, but not to their GDP-bound forms. The right two lanes show positive binding (between GTP-bound KRAS-G12D and RAF-RBD) and negative binding (between GDP-bound KRAS-G12D and RAF-RBD) controls. Bottom, purified KRAS protein. **j**–**m**, Lysates from serum or EGF-treated or EGF-untreated KRAS-wild-type H1703 cells were immunoprecipitated with antibody to RanGAP1 or mock IgG, followed by immunoblotting with antibody to KRAS or RanGAP1. Serum and EGF treatment induces ERK activity (measured by pERK-T202/Y204; **j** and **l**) and complex formation between KRAS and RanGAP1 (**k** and **m**). **n**–**p**, Overexpressing dominant-negative mutant KRAS-S17N (**n**) reduces complex formation between KRAS and RanGAP1 (**o**) and between KRAS and BRAF to a similar degree (**p**). Lysates from NCI-H23 cells overexpressing KRAS-G12C or KRAS-S17N were immunoprecipitated with antibody to RanGAP1 or mock IgG, followed by immunoblotting with antibody to KRAS or RanGAP1. Two independent experiments were performed for each image with similar results. Source data

If the KRAS–RanGAP1 complex contributes to XPO1-dependent protein export, KRASi might prevent the cytoplasmic release of the nuclear protein cargo, whereas increased KRAS activity would promote protein cargo release. Consistent with this hypothesis, KRASi of NCI-H23 cells resulted in increased binding of XPO1 and EZH2 to NUP358 (Fig. 2f–h), and overexpressed mutant KRAS-G12C decreased this binding (Fig. 2i–m). Furthermore, if the KRAS–RanGAP1 complex regulates the cytoplasmic release of the nuclear protein cargo by the hypothesized mechanism, KRASi would be expected to increase cytoplasmic Ran•GTP, whereas KRAS overexpression would decrease it. Indeed, KRASi increased cytoplasmic Ran•GTP (Fig. 3a,b), whereas overexpressing KRAS-G12D decreased it (Fig. 3c–f).

**Extended Data Fig. 4:**
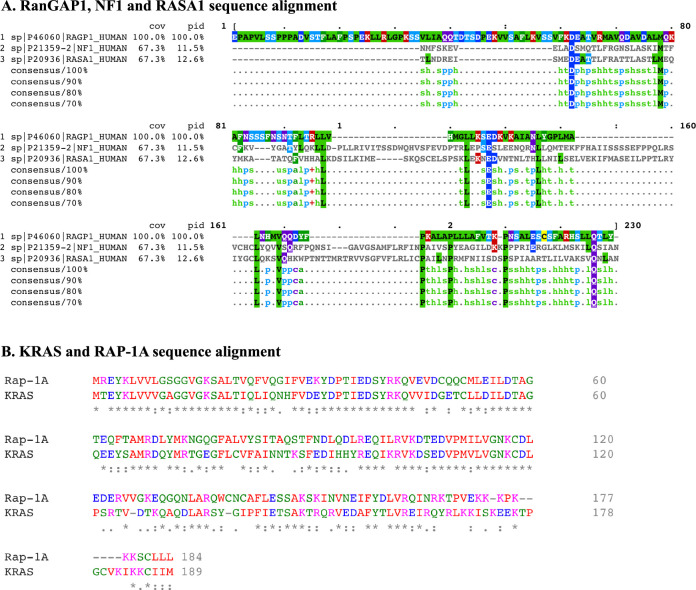
Sequence alignment of the indicated proteins. Multiple sequence alignment using CLUSTAL Omega (1.2.4, https://www.ebi.ac.uk/Tools/msa/clustalo/). **a** Protein sequence alignment among RanGAP1, NF1, and RASA1. **b** Protein sequence alignment between KRAS and RAP-1A.

Additional biochemical analyses confirmed the direct interaction between KRAS and RanGAP1. First, we demonstrated a stable complex between GFP-tagged mutant KRAS-G12C or KRAS-G12D and glutathione *S*-transferase (GST)-tagged full-length RanGAP1 (amino acids 1–587) or its catalytic domain (amino acids 1–416; Extended Data Fig. 3k–n). The homology between the catalytic domains of two well-known RAS-GAPs (RASA1 and NF-1) and the analogous domain of RanGAP1 (Extended Data Fig. 4a) support these results. Furthermore, purified wild-type KRAS or mutant KRAS-G12D loaded with either GTP or GDP and incubated with purified RanGAP1 protein confirmed that only the GTP-loaded KRAS bound RanGAP1 (Fig. 3g–i). As positive and negative specificity controls, respectively, purified RAF-RAS binding domain (RAF-RBD) specifically bound to GTP-loaded KRAS (Fig. 3i, right), whereas purified CUB domain containing protein 1 (CDCP1) did not (Fig. 3h).

Treatment of cells with serum or epidermal growth factor (EGF) are two ways to increase RAS•GTP, while a dominant-negative (DN) RAS mutant (KRAS-S17N) can decrease RAS•GTP^21–23^. The above hypothesis suggested that serum or EGF treatment would increase the level of KRAS–RanGAP1 complex formation in cells, while a DN RAS mutant would have the opposite effect. Consistent with this hypothesis, stimulation of wild-type KRAS H1703 cells with serum or EGF increased KRAS–RanGAP1 complex formation (Fig. 3j–m), while transfection of DN KRAS-S17N mutant in NCI-H23 cells reduced KRAS–RanGAP1 and KRAS–BRAF complex formation to a similar degree (Fig. 3n–p).

**Fig. 4:**
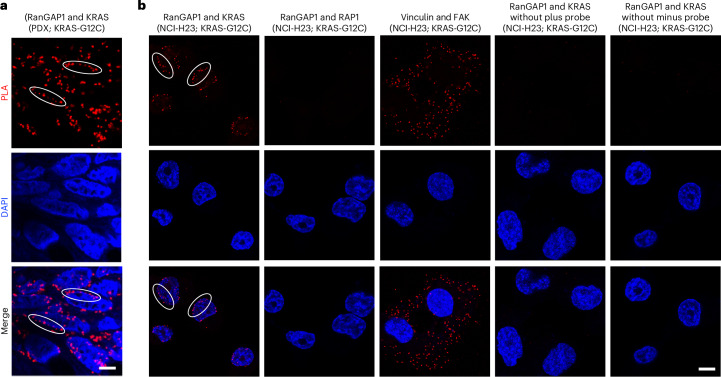
KRAS and RanGAP1 form a perinuclear complex in PDX sections and NCI-H23 cells. **a**, PDX tumor sections with KRAS-G12C showed perinuclear PLA signals of colocalization of RanGAP1 and KRAS. Tumor sections were immunostained with antibodies to RanGAP1 and KRAS. DAPI (blue) was used to stain the nuclei. White oval outlines indicate some of the red perinuclear signals; scale bar, 5 µm. **b**, Perinuclear PLA colocalization signal between RanGAP1 and KRAS in NCI-H23 cells (first column). The wider cell distribution of the PLA colocalization signals between vinculin and FAK (third column) was distinct from that between RanGAP1 and KRAS (first column), while there was no PLA signal between RanGAP1 and RAP1 GTPase (second column). There was no PLA signal detected when plus probe (middle columns) or minus probe was omitted (fourth and fifth images); scale bar, 10 µm. Two independent experiments were performed for each image with similar results.

**Extended Data Fig. 5:**
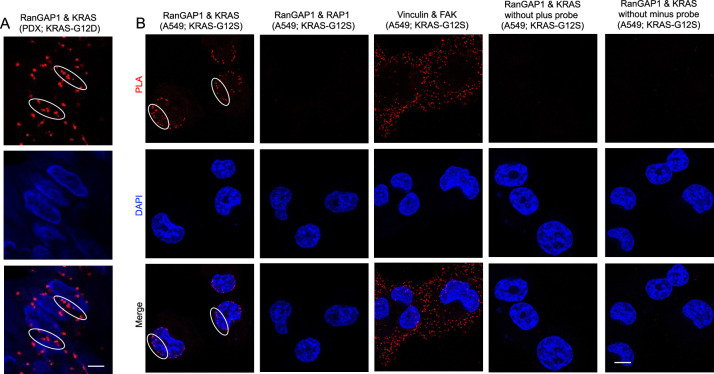
KRAS and RanGAP1 form a perinuclear complex in PDX and A549 cells. **a** PDX tumor sections with KRAS-G12D showed perinuclear PLA signals of colocalization of RanGAP1 and KRAS. Tumor sections were immunostained with RanGAP1 and KRAS antibodies. DAPI was used to stain nuclei (blue). White oval outlines indicate red perinuclear signals. Scale bar = 5 µm. **b** Perinuclear PLA colocalization signal between RanGAP1 and KRAS were also observed in A549 cells (first panel). The wider distribution of the PLA colocalization signal between Vinculin and FAK (third panel) was distinct from that between RanGAP1 and KRAS (first panel), while there was no PLA signal between RanGAP1 and RAP1 (second panel). There was no PLA signal detected when plus probe (middle panels) or minus probe was omitted (fourth and fifth panels). Scale bar = 10 µm. Two independent experiments were performed for each image with similar results.

We used two approaches to identify the KRAS–RanGAP1 complex in tumor cells. One approach was a proximity ligation assay (PLA), which produced a positive PLA colocalization signal for RanGAP1 and KRAS in sections from a patient-derived xenograft (PDX) with mutant KRAS-G12C (Fig. 4a), and the NCI-H23 line with mutant KRAS-G12C (Fig. 4b, first image). Many of the red colocalization signals appeared to be perinuclear (Fig. 4a,b, first image, white ovals), whereas some appeared to be in the cytoplasm. There was no PLA signal between RanGAP1 and the RAP1 GTPase (Fig. 4b, second image, and Extended Data Fig. 4b), the RAS-related protein that is closest to RAS^24^, or with other negative controls (Fig. 4b, fourth and fifth images). PLA colocalization of the focal adhesion proteins vinculin and FAK was included as a positive control, where colocalization differs from KRAS and RanGAP1 (Fig. 4b, third image). Similar perinuclear colocalization between KRAS and RanGAP1 was seen in another PDX section with KRAS-G12D and the A549 (KRAS-G12S) line (Extended Data Fig. 5a,b).

**Fig. 5:**
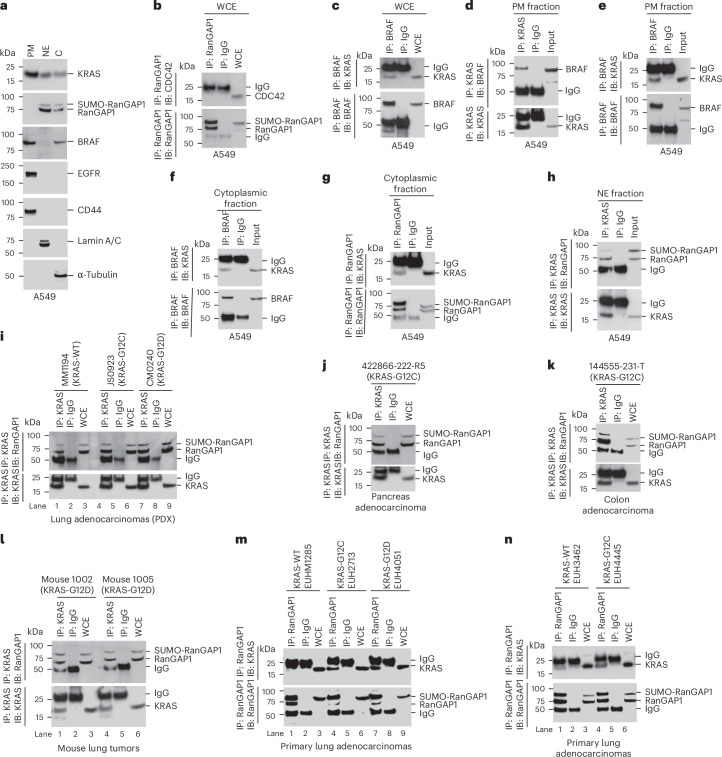
Cell fractionations for PM, NE and cytoplasmic fractions. The KRAS–RanGAP1 complex occurs in many tumors, including in primary human lung cancer, PDXs from lung, pancreas and colorectal cancer and a KRAS-induced mouse lung cancer model. **a**, A549 cells were fractionated for PM, NE and cytoplasmic fractions, and the purity of each fraction was verified by the expression of specific marker proteins, for example, EGFR and CD44 for the PM, lamin A/C for the NE and α-tubulin for the cytoplasm. KRAS is present in all three fractions, RanGAP1 is present only in NE and cytoplasmic fractions, and BRAF is present only in the PM and cytoplasmic fractions. **b**, Lysates from A549 cells were immunoprecipitated with antibody to RanGAP1 or mock IgG, followed by immunoblotting with antibody to CDC42 or RanGAP1. **c**–**h**, Lysates from the indicated fractions were immunoprecipitated with antibody to BRAF, KRAS or RanGAP1 or mock IgG, followed by immunoblotting with antibody to KRAS, BRAF or RanGAP1; Input, indicated fraction. KRAS formed a complex with BRAF in the whole-cell extract (**c**), PM (**d** and **e**) and cytoplasmic fractions (**f**), and KRAS formed a complex with RanGAP1 in the cytoplasmic (**g**) and NE (**h**) fractions. **i**–**n**, Lysates from the indicated samples were immunoprecipitated with antibody to KRAS or RanGAP1 or mock IgG, followed by immunoblotting with antibody to RanGAP1 or KRAS. KRAS–RanGAP1 protein complexes were identified in PDXs from lung adenocarcinoma (**i**), pancreas adenocarcinoma (**j**) and colon adenocarcinoma (**k**); KRAS-inducible lung adenocarcinoma in mice (**l**) and primary human lung adenocarcinoma (**m** and **n**). Two independent experiments were performed for each image with similar results. Source data

**Extended Data Fig. 6:**
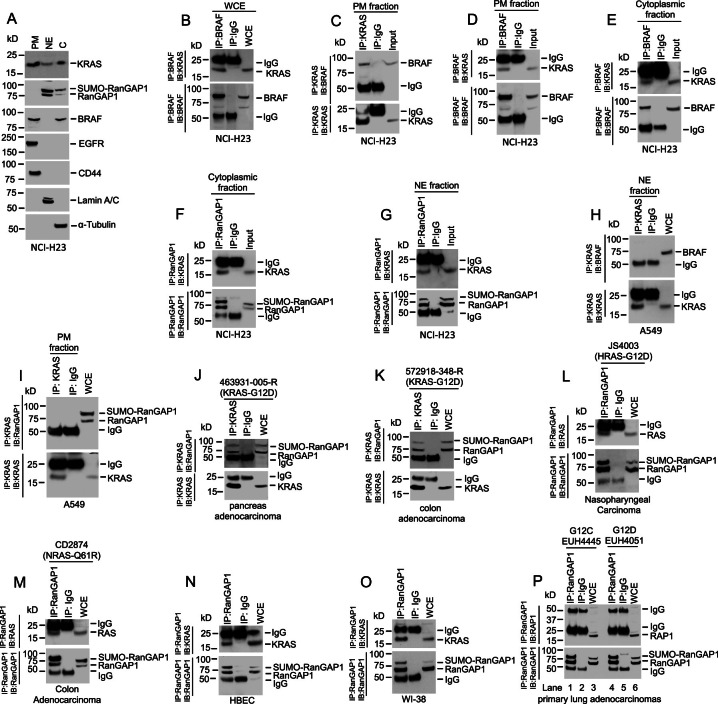
Cell fractionations for plasma membrane (PM), nuclear envelope (NE), and cytoplasmic (C) fractions. The RAS:RanGAP1 complexes are present in many tumor types and non-tumorigenic lines. **a** NCI-H23 cells were fractionated for PM, NE, and cytoplasmic fractions whose purity was verified by specific marker proteins, for example, EGFR and CD44 for the PM, lamin A/C for the NE, and α-tubulin for the cytoplasmic marker protein. KRAS is present in all three fractions, RanGAP1 is present only in NE and cytoplasmic fractions, while BRAF is present only in PM and cytoplasmic fractions. **b-i** Lysates from indicated fractions were IP with antibodies to BRAF, KRAS, RanGAP1, or mock IgG, followed by IB with antibodies to KRAS, BRAF, or RanGAP1. WCE, whole cell extract, Input, indicated purified fraction. KRAS formed a complex with BRAF in the WCE (**b**), PM (**c,d**), and cytoplasmic fraction (**e**). KRAS formed a complex with RanGAP1 in cytoplasmic fraction (**f**) and NE (**g**). **h** As BRAF is not present in NE (**a**), there was no complex formation between KRAS and BRAF in NE fraction. **i** Since RanGAP1 is not present in PM (**a**), there was no complex formation between KRAS and RanGAP1 in PM fraction. **j-p** Lysates from indicated samples were IP with antibodies to KRAS, RanGAP1, or mock IgG, followed by IB with antibodies to RanGAP1 or KRAS. WCE, whole cell extract. KRAS:RanGAP1 protein complexes were identified in: PDX’s from pancreas adenocarcinoma (**j**), PDX’s from colon adenocarcinoma (**k**), and PDX’s from HRAS mutant Nasopharyngeal Carcinoma (**l**), PDX’s from NRAS mutant colon adenocarcinoma (**m**), non-transformed HBEC line (**n**), non-immortalized, non-transformed WI-38 fibroblasts (**o**). In contrast to the positive results of complex formation between KRAS and RanGAP1 in primary human lung cancer shown in Fig. 5 l,m, the complex formation was not detected between RanGAP1 and the RAP1 (**p**). Two independent experiments were performed for each image, with similar results. Source data

As a second approach for identifying the KRAS–RanGAP1 complex in cells, we made three cell fractions from A549 and NCI-H23 cells, plasma membrane (PM), cytoplasmic and nuclear envelope (NE), and analyzed them for the presence of complexes between KRAS and either RanGAP1 or BRAF (Fig. 5a–g and Extended Data Fig. 6a–g). Specific markers used were EGF receptor (EGFR) and CD44 for the PM, α-tubulin for the cytoplasm and lamin A/C for the NE (Fig. 5a and Extended Data Fig. 6a). KRAS was present in all three fractions, RanGAP1 was only in the NE and cytoplasmic fractions, and BRAF was only in the PM and cytoplasmic fractions (Fig. 5a and Extended Data Fig. 6a). There was no complex formation between RanGAP1 and a non-RAS GTPase CDC42 in a whole-cell extract of A549 cells (Fig. 5b). Although KRAS formed a complex with BRAF in the whole-cell extract, PM and cytoplasmic fractions (Fig. 5c–f and Extended Data Fig. 6b–e), the KRAS–RanGAP1 complex was only present in the cytoplasmic and NE fractions (Fig. 5g,h and Extended Data Fig. 6f,g). There was no KRAS–BRAF complex in the NE fraction (Extended Data Fig. 6h) or KRAS–RanGAP1 complex in the PM fraction (Extended Data Fig. 6i).

### RAS–RanGAP1 complexes are present in many tumor types and nontumorigenic lines

The KRAS–RanGAP1 complex occurs in many settings. We identified this complex in every PDX sample evaluated, including PDXs from lung, pancreatic and colorectal cancers with mutant KRAS, mutant HRAS or mutant NRAS and from the HBEC line as well as from the WI-38 nonimmortalized nontransformed human lung fibroblast line (Fig. 5i–k and Extended Data Fig. 6j–o). Consistent with our observation that the GTP-bound form of KRAS preferentially binds RanGAP1, as complex formation with wild-type KRAS appeared to be lower than with mutant KRAS (Fig. 5i, compare lane 1 with lanes 4 and 7). The complex was also found in lung tumors from a widely used conditional mutant KRAS-G12D mouse model^25^ (Fig. 5l). Perhaps most important, the complex was present in primary human lung adenocarcinomas with wild-type KRAS, KRAS-G12C mutant and KRAS-G12D mutant, with greater binding observed in the mutants (Fig. 5m,n), while complex formation was not detected between RanGAP1 and RAP1 (Extended Data Fig. 6p).

### HRAS, NRAS and KRAS bind RanGAP1, with the most efficient binding requiring RAS farnesylation

**Fig. 6:**
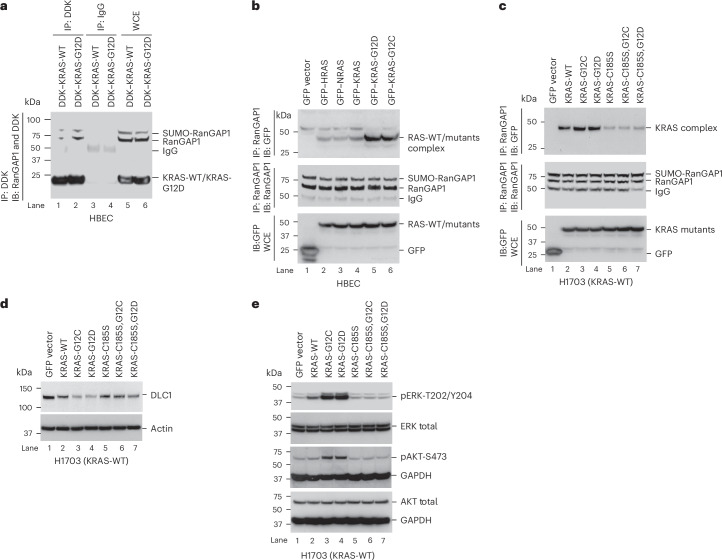
RanGAP1 forms a complex with all three RAS proteins, which is enhanced by RAS farnesylation. **a**, RanGAP1 bound more efficiently to mutant KRAS-G12D than to wild-type KRAS. **b**, The RanGAP1–RAS complex formed with similar efficiency with wild-type HRAS, NRAS and KRAS and was more efficient with mutant KRAS-G12C and KRAS-G12D. **c**–**e**, H1703 cells were stably transfected with the indicated KRAS mutant and analyzed for several parameters. The RanGAP1–KRAS complex was most efficient with farnesylated KRAS, which is associated with the greatest decrease in DLC1 protein expression. Formation of the RanGAP1–KRAS complex was greater with KRAS-G12C and KRAS-G12D mutants (**c**, lanes 3 and 4) than with isogenic farnesylation-deficient C185S mutants (**c**, lanes 6 and 7), which was correlated with a greater reduction in DLC1 protein expression (**d**, lanes 3 and 4) than observed with the farnesylation-deficient C185S mutants (**d**, lanes 6 and 7). The KRAS-G12C and KRAS-G12D mutants have the highest activation of ERK and AKT (**e**, lanes 3 and 4), as measured by pERK-T202/Y204 and pAKT-S473 expression, respectively. Two independent experiments were performed for each image with similar results. Source data

To confirm that increased abundance of KRAS•GTP induces more KRAS–RanGAP1 complex formation, the HBEC line was transfected with DDK-tagged wild-type KRAS or KRAS-G12D mutant; complex formation was found to be greater with the mutant KRAS (Fig. 6a). To determine whether the proteins encoded by the three *RAS* genes (*HRAS*, *KRAS* and *NRAS*) bound RanGAP1 with similar efficiency, each wild-type RAS protein tagged with the same epitope (GFP) was immunoprecipitated with anti-RanGAP1 and immunoblotted with anti-GFP, resulting in similar binding signals (Fig. 6b, compare lanes 2, 3 and 4). RanGAP1 binding was stronger with mutant KRAS-G12C and mutant KRAS-G12D than with wild-type KRAS (Fig. 6b, compare lane 4 with lanes 5 and 6).

We also discovered that farnesylation of the KRAS protein, which is dependent on its C-terminal cysteine and increases membrane association^26^^,27^, is required for more efficient RanGAP1 binding and downstream signaling. To examine this parameter, we compared wild-type and mutant KRAS protein with or without mutation of the KRAS C-terminal cysteine (C185S) in the H1703 line. Maximal KRAS–RanGAP1 binding (Fig. 6c) and reduced DLC1 protein expression (Fig. 6d) were detected with mutant KRAS, whereas the C185S mutant was associated with reduced binding (Fig. 6c) and increased DLC1 protein expression (Fig. 6d). As expected, the C185S mutants did not induce ERK or AKT activation, whereas their isogenic wild-type counterparts did (Fig. 6e).

### Biological importance of the KRAS–RanGAP1 complex and XPO1-dependent activity

To explore the biological relevance of our KRAS and XPO1 findings, we used three experimental models. Two were the mutant KRAS cell lines NCI-H23 (KRAS-G12C) and A549 (KRAS-G12S). Both cell lines were used to study the ability of various pharmacologic inhibitors to interfere with anchorage-independent growth and tumor xenograft growth. The third was the conditional mutant *Kras*^G12D^/*Trp53* mouse model^25^, where we examined the impact of several combinations of inhibitors.

**Fig. 7:**
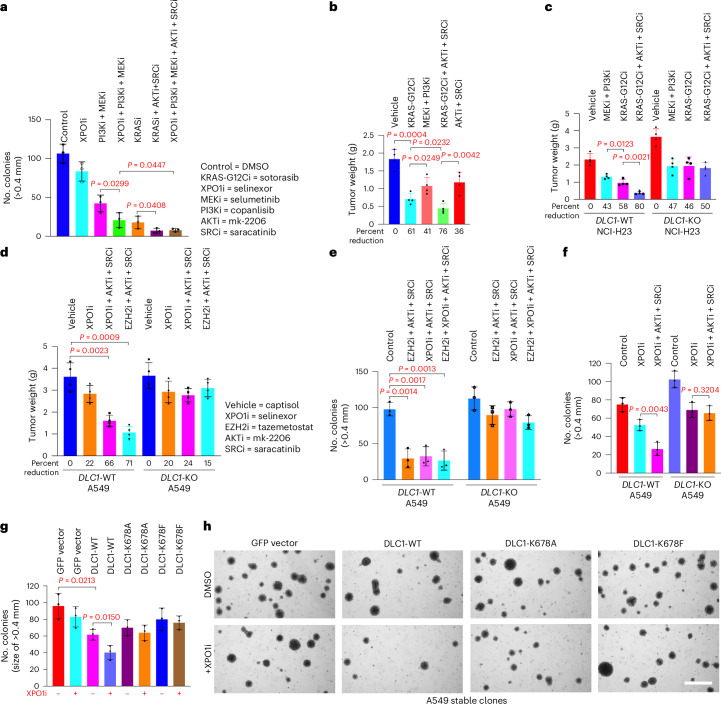
The combination of XPO1i + MEKi + PI3Ki inhibits cell growth to the same degree as KRASi, facilitated by DLC1-dependence. **a**, Quantitation of cell colonies (>0.4 mm) in response to the indicated treatment. Bar graphs represent mean, and error bars represent s.d.; *n* = 3. Combined XPO1i + PI3Ki + MEKi showed similar inhibition as KRASi. KRASi + AKTi + SRCi showed greater inhibition than KRASi; *P* = 0.0299 for PI3Ki + MEKi versus XPO1i + PI3Ki + MEKi, *P* = 0.0408 for KRAS-G12Ci versus KRAS-G12Ci + AKTi + SRCi, and *P* = 0.0447 for XPO1i + PI3Ki + MEKi versus XPO1i + PI3Ki + MEKi + AKTi + SRCi. **b**, In NCI-H23 xenografts, KRASi + AKTi + SRCi had the highest antitumor activity, followed by KRASi, with PI3Ki + MEKi having the lowest activity. The numbers below each graph represent percent reduction in tumor weight for each treatment group compared to vehicle; *P* = 0.0004 for vehicle versus KRAS-G12Ci, *P* = 0.0249 for KRAS-G12Ci versus MEKi + PI3Ki, *P* = 0.0232 for KRAS-G12Ci versus KRAS-G12Ci + AKTi + SRCi, and *P* = 0.0042 for KRAS-G12Ci + AKTi + SRCi versus AKTi + SRCi. **c**, Differences in treatment responses to various inhibitors seen in the *DLC1*-WT parental line were abrogated in a *DLC1*-KO line; *P* = 0.0123 for MEKi + PI3Ki versus KRAS-G12Ci and *P* = 0.0021 for KRAS-G12Ci versus KRAS-G12Ci + AKTi + SRCi. **d**, In A549 xenografts, treatment with AKTi + SRCi plus XPO1i or EZH2i had similar antitumor activity. In **b**–**d**, individual and average tumor weight are shown. The bar graphs represent mean, error bars represent s.d.; *n* = 4; *P* = 0.0023 for vehicle versus XPO1i + AKTi + SRCi and *P* = 0.0009 for vehicle versus EZH2i + AKTi + SRCi. **e**, Quantitation of colonies after treatment. The four-drug combination was not more inhibitory than the three-drug combination without XPO1i or EZH2i; *P* = 0.0014 for control versus EZH2i + AKTi + SRCi, *P* = 0.0017 for control versus XPO1i + AKTi + SRCi, and *P* = 0.0013 for control versus EZH2i + XPO1i + AKTi + SRCi. **f**, Quantitation of colonies after treatment. The three-drug combination of XPO1i + AKTi + SRCi was more inhibitory than XPO1i; *P* = 0.0043 for XPO1i versus XPO1i + AKTi + SRCi in *DLC1*-WT and *P* = 0.3204 for XPO1i versus XPO1i + AKTi + SRCi in *DLC1*-KO. **g**,**h**, Quantitation of colonies after treatment (**g**), as shown in photomicrographs (**h**); scale bar, 2 mm. For **e**–**g**, bar graphs represent mean, and error bars represent s.d.; *n* = 3. Anchorage-independent growth in response to XPO1i in stably transfected DLC1 mutants. For statistical analyses in **a**–**g**, parametric unpaired one-tailed *t*-tests with Welch’s correction were performed; *P* = 0.0213 for GFP vector versus *DLC1*-WT-transfected cells and *P* = 0.0150 for untreated versus XPO1i-treated *DLC1*-WT-transfected cells. Source data

**Extended Data Fig. 7:**
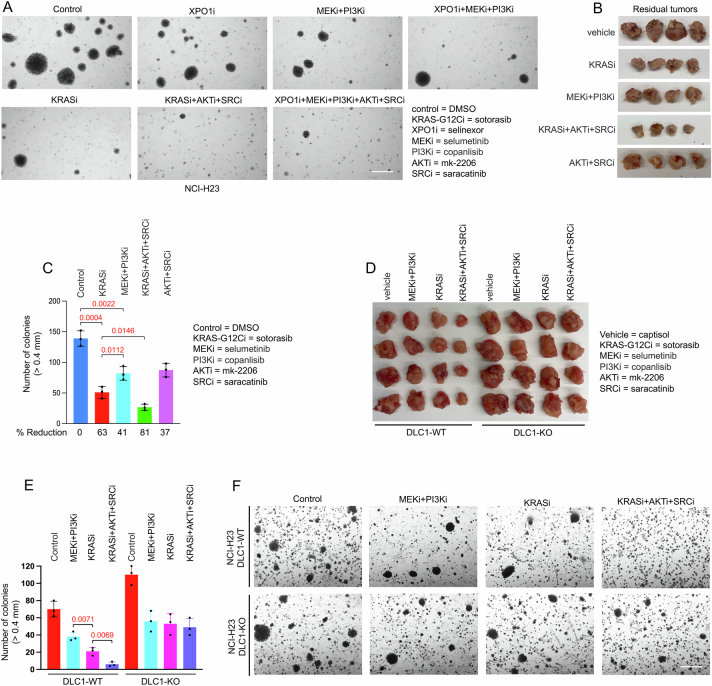
The combined inhibition of XPO1, MEK, and PI3K inhibits cell growth to the same degree as KRAS inhibition. The combined inhibition of KRAS, AKT, and SRC has the highest antitumor activity; DLC1 expression makes a critical contribution to this antitumor activity. **a** Photomicrographs of colonies quantified in Fig. 7a. Scale bar = 2 mm. Anchorage-independent colonies growth in responses to treatment with the KRAS inhibitor sotorasib alone or in the combination of the indicated drugs treatment. The combined inhibition of XPO1, PI3K, and MEK have similar colonies growth inhibition as KRAS inhibition. The combined inhibition of KRAS, AKT, and SRC had greater colonies growth inhibition compared to KRAS inhibition alone. **b** Xenograft tumors after 3 weeks of treatment with the indicated inhibitors quantified in Fig. 7b. **c** Quantitation of agar colonies (>0.4 mm) after the indicated drugs treatment. Bar graphs represent mean value and error bars represent SD. N = 3 independent experiments. p = 0.0004 for control versus KRAS-G12Ci, p = 0.0022 for control versus MEKi+PI3Ki, p = 0.0112 for KRAS-G12Ci versus MEKi+PI3Ki, and p = 0.0146 for KRAS-G12Ci versus KRAS-G12Ci+AKTi+SRCi. **d** Excised xenograft tumors after treatment with the indicated inhibitors. In the NCI-H23 xenograft tumors, combined inhibition of KRAS, AKT, and SRC has the highest antitumor activity in DLC1 expressed tumors. Most of the antitumor activity was attributable to DLC1 protein expression, as the isogenic DLC1-KO line was much less susceptible to the three-drug combination. **e,f** Quantitation (**e**) of agar colonies (>0.4 mm) after the indicated drugs treatment, as photomicrographs of colonies shown (**f**). In figure E, bar graphs represent mean value and error bars represent SD. N = 3 independent experiments. p = 0.0071 for MEKi+PI3Ki versus KRAS-G12Ci and p = 0.0069 for KRAS-G12Ci versus KRAS-G12Ci+AKTi+SRCi. In Figure F, hotomicrographs of anchorage-independent colonies growth in responses to treatment with KRAS inhibitor sotorasib and with the indicated inhibitors in NCI-H23 parental line and isogenic DLC1-KO line. Scale bar = 2 mm. For the statistical analysis for Figures C and E, parametric unpaired one tailed *t*-test with Welch’s correction was performed using Prism software, and no adjustments were made for multiple comparisons. Source data

If the effect of the KRAS–RanGAP1 complex is biologically relevant, we hypothesized that mutant KRASi would restrict growth to a greater degree than PI3Ki + MEKi (by copanlisib and selumetinib, respectively), whereas the addition of XPO1i (selinexor) to this combination would inhibit growth similar to KRASi. Using anchorage-independent growth of the NCI-H23 line, we confirmed this possibility; KRASi inhibited growth to a greater degree than PI3Ki + MEKi, whereas XPO1i + PI3Ki + MEKi was similar to KRASi (Fig. 7a, columns 3–5, and Extended Data Fig. 7a). The growth inhibitory activity of XPO1i was less than that of PI3Ki + MEKi (Fig. 7a, columns 2 and 3, and Extended Data Fig. 7a).

Our previous publication had shown that although EZH2 inhibition (EZH2i; tazemetostat) stabilized the DLC1 protein^4^, it had only a marginal effect on the tumor suppressor activity of DLC1. This reduced DLC1 activity in human lung cancer was attributable to AKT and SRC kinase activities, both of which reduce the tumor suppressor activity of DLC1 via direct phosphorylation of specific tyrosines by SRC and specific serines by AKT^28^. However, the combined inhibition of AKT inhibition (AKTi) + SRC inhibition (SRCi; by mk-2206 and saracatinib, respectively) can inhibit these phosphorylation activities and reactivate the tumor suppressor activity of DLC1 stabilized by EZH2i^4^. As the current results indicated that the impact of KRASi or XPO1i on DLC1 expression was similar to EZH2i, we experimentally verified that AKTi + SRCi would augment the growth inhibitory activities of KRASi alone and XPO1i alone (Fig. 7a, columns 4–7, and Extended Data Fig. 7a).

We extended many of these findings to growth inhibition of tumor xenografts and anchorage-independent growth assays of the NCI-H23 cell line (Fig. 7b and Extended Data Fig. 7b,c). Specifically, we confirmed that KRASi was more potent than PI3Ki + MEKi and that the growth inhibitory activity of KRASi + SRCi + AKTi was more potent than KRASi alone. In addition, we constructed an isogenic cell line from which the *DLC1* gene had been disrupted (*DLC1*-KO) by CRISPR–Cas9 technology. Although the percentage of growth inhibition by PI3Ki + MEKi was similar in both the parental (*DLC1*-WT) and *DLC1*-KO line, the degree of KRASi or KRASi + SRCi + AKTi on growth inhibition was less in the *DLC1*-KO line than in the *DLC1*-WT line (Fig. 7c and Extended Data Fig. 7d). These results indicate that DLC1 contributes to some of the growth inhibitory activity of KRASi and KRASi + SRCi + AKTi in *DLC1*-WT cells, but not to the inhibition by PI3Ki + MEKi. Similar results were seen when these combinations of inhibitors were used in an anchorage-independent growth assay (Extended Data Fig. 7e,f).

**Extended Data Fig. 8:**
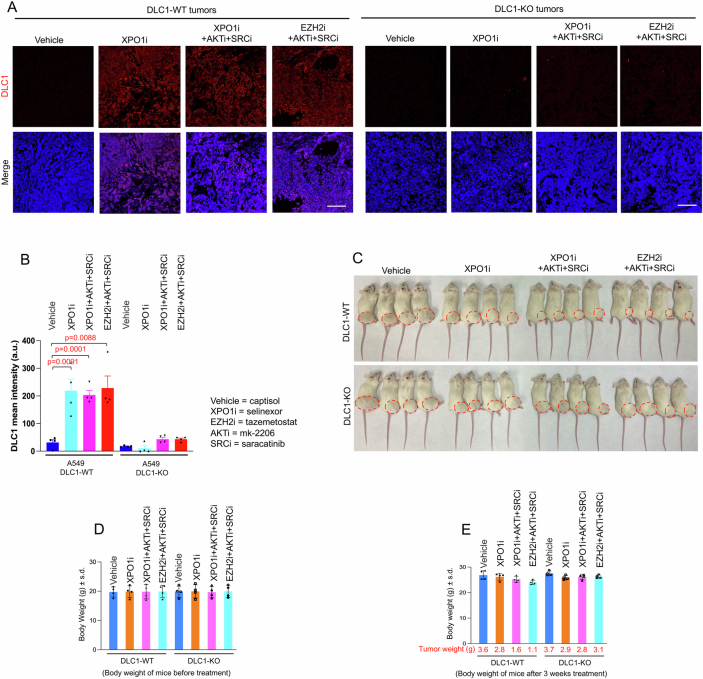
Selinexor and tazemetostat treatment induce DLC1 protein expression in tumors. Treatment with the combination of three-drugs is not associated with changes in mouse coat appearance or weight loss. **a** Sections from selinexor and tazemetostat treated tumors in Fig. 7d and Extended Data Fig.  8C were immunostained with antibodies to DLC1 (red) and DAPI was used to stain nuclei (blue). Tumor treated with selinexor alone or in combination with mk-2206 and saracatinib expressed higher levels of DLC1 protein than the vehicle control tumors. Scale bar, 100 μm. However, there were no upregulation of DLC1 protein in DLC1-KO line. **b** Quantification of DLC1 mean intensity in arbitrary units. Bar graphs represent mean value and error bars represent SD. N = 4 tumor sections per group. For the statistical analyses for Figure B, parametric unpaired one-tailed *t*-test with Welch’s correction was performed using Prism software, and no adjustments were made for multiple comparisons. p = 0.0091 for vehicle versus XPO1i, p = 0.0001 for vehicle versus XPO1i+AKTi+SRCi, and p = 0.0088 for vehicle versus EZH2i+AKTi+SRCi. **c** Treatment of tumor bearing mice with three-drug combination did not show visible side effects, such as change in coat appearance or weight loss unrelated to a decrease in tumor weight. N = 4 mice per group. **d,e** Graphs show average body weight of mice before treatment (**d**) and after three weeks treatment (**e**) in all groups. In Figure D and E, bar graphs represent mean value of mice weight for each group, and error bars represent SD. N = 4 mice per group for both D and E. The numbers at the bottom of panel E represent the average tumor weight. Much of the change in weight is attributable to the weight of the tumor. Source data

We used the A549 cell line and its isogenic *DLC1*-KO line^4^ to directly demonstrate that EZH2i and XPO1i were similar biologically in tumor xenograft and anchorage-independent growth assays. In both assays, the results indicated that the growth inhibitory activities of EZH2i + SRCi + AKTi or XPO1i + SRCi + AKTi were similar in parental *DLC1*-WT cells and that part of the activity of the inhibitor combinations depended on DLC1 (Fig. 7d–f and Extended Data Fig. 8a,b). For the mice used in the xenograft assay, there were no obvious side effects, such as weight loss, from either three-drug combination (Extended Data Fig. 8c–e).

We previously determined that the EZH2-dependent decrease in DLC1 expression was attributable to methylation of DLC1-K678 by cytoplasmic EZH2 and its subsequent degradation by ubiquitination and the proteasome^4^. To more directly establish a role for EZH2-dependent methylation of DLC1 in the inhibitory activities of XPO1, we made stable transfectants of the A549 line by expressing wild-type DLC1, DLC1-K678A (methylation deficient) or DLC1-K678F (methylation mimetic) and treated them with XPO1i (Fig. 7g,h). Although the colony growth of wild-type DLC1 transfectants was inhibited by XPO1i, the colony growth of the two methylation-resistant mutant transfectants was not affected by XPO1i.

**Fig. 8:**
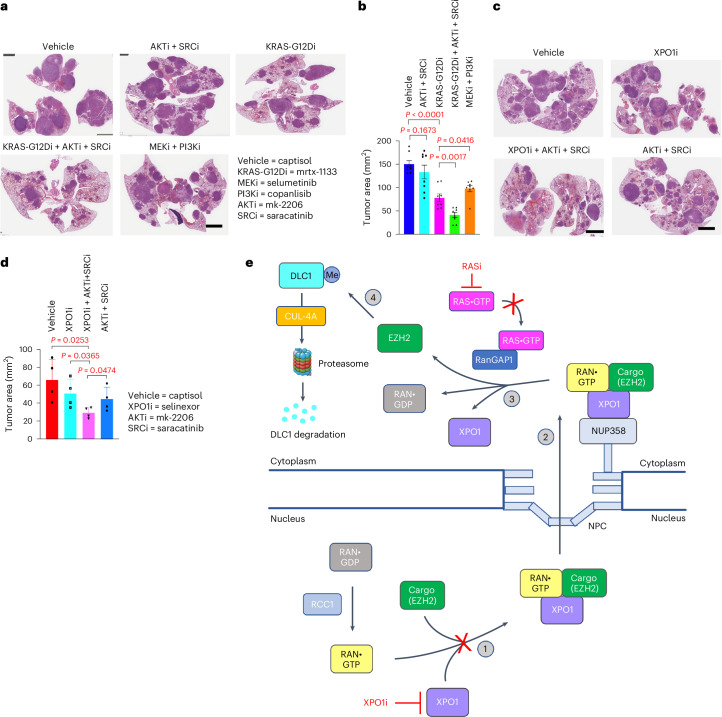
KRASi or XPO1i cooperates with the inhibition of AKT kinase and SRC kinase in antitumor activity. **a**,**b**, In mouse lung tumors (**a**) induced by KRAS-G12D activation and p53 inactivation, the combination of mrtx-1133 + mk-2206 + saracatinib showed greater antitumor activity than mrtx-1133 alone or the combination of mk-2206 + saracatinib. Mrtx-1133 had greater antitumor activity than the selumetinib + copanlisib combination; scale bar, 4 mm. Bar graphs in **b** represent mean values of residual tumor area, and error bars represent s.d.; *n* = 8 mice per group; *P* = 0.1673 for vehicle versus AKTi + SRCi, *P* = 0.0001 for vehicle versus KRAS-G12Di, *P* = 0.0017 for KRAS-G12Di versus KRAS-G12Di + AKTi + SRCi, and *P* = 0.0416 for KRAS-G12Di versus MEKi + PI3Ki. **c**,**d**, The combination of selinexor + mk-2206 + saracatinib had greater antitumor activity than selinexor alone or the combination of mk-2206 + saracatinib (**c**); scale bar, 4 mm. Bar graphs in **d** represent mean values of residual tumor area, and error bars represent s.d.; *n* = 4 mice per group. For the statistical analyses for **b** and **d**, parametric unpaired one-tailed *t*-tests with Welch’s correction were performed using Prism software, and no adjustments were made for multiple comparisons; *P* = 0.0253 for vehicle versus XPO1i + AKTi + SRCi, *P* = 0.0365 for XPO1i versus XPO1i + AKTi + SRCi, and *P* = 0.0474 for XPO1i + AKTi + SRCi versus AKTi + SRCi. **e**, Model summarizing the key noncanonical steps identified in this study. (1) Formation of a trimeric protein complex (Ran•GTP–XPO1–cargo) in the nucleus, which can be abrogated by XPO1i. (2) The trimeric complex that is exported through the NPC becomes associated with NUP358, which is part of the cytoplasmic face of the NPC. (3) RAS•GTP and RanGAP1 form a complex that facilitates the hydrolysis of Ran•GTP to Ran•GDP and releases the cargo into the cytoplasm; RASi can prevent this step. (4) EZH2, which is a key protein cargo identified in this study, methylates DLC1, which can then be ubiquitinated and subjected to proteasome-dependent degradation. Source data

We also performed a limited number of studies in the conditional *Kras*^G12D^/*Trp53* mouse lung cancer model, with activation of the endogenous KRAS-G12D mutation and disruption of p53 by inhalation of an adenovirus vector encoding the Cre recombinase (Fig. 8a–d). The results were similar to those found in the NCI-H23 and A549 bioassays. Specifically, KRASi alone (by the KRAS-G12D-specific inhibitor mrtx-1133) was more potent than AKTi + SRCi, KRASi showed even greater growth inhibition when combined with AKTi + SRCi, and XPO1i + AKTi + SRCi was more potent than XPO1i alone.

## Discussion

In this study, we identified a previously undescribed complex between RAS and RanGAP1 that mediates a noncanonical RAS-dependent function that lies within the XPO1 nuclear protein export pathway. The cytoplasmic export of EZH2, which methylates the DLC1 tumor suppressor protein and decreases its half-life^4^, is one critical component of this pro-oncogenic function. The main findings, which are summarized in Fig. 8e, have implications for cancer development, maintenance and treatment.

We determined that most of the previously identified increase in cytoplasmic EZH2 expression in lung cancer^4^ is derived from nuclear EZH2 via XPO1-dependent export (Fig. 8e, step 1). XPO1i leads to an increase in the steady-state level of DLC1 protein expression, and it substitutes functionally for the EZH2i that we reported previously^4^. In NSCLC lines, the combination of XPO1i together with AKTi and SRCi, which reactivate the RhoGAP and tumor suppressor activities of DLC1 (ref. ^28^), potently inhibited tumor growth. Analysis of isogenic cell lines in which *DLC1* had been disrupted indicated that DLC1 made an important contribution to the growth inhibitory activity of XPO1i + AKTi + SRCi treatment, although each inhibitor has multiple targets that, with the notable exception of DLC1, are largely nonoverlapping.

Although *RAS* has not been directly implicated previously in nucleocytoplasmic shuttling, we found that increased *KRAS* activity phenocopied increased *XPO1*, including an increase in cytoplasmic EZH2 and survivin and a decrease in DLC1 (Fig. 8e, steps 3 and 4), whereas KRASi had the opposite effects. However, KRASi inhibited XPO1 function at a step downstream from XPO1i, as KRASi did not affect XPO1–EZH2 complex formation, unlike XPO1i.

The RAS-dependent activity results from a previously undescribed complex between KRAS•GTP and RanGAP1, which regulates the Ran GTPase, a master regulator of nucleocytoplasmic shuttling^3^ (Fig. 8e, step 3). When Ran is bound by GDP, it mediates the import of proteins from the cytoplasm to the nucleus, and, when bound to GTP, it mediates the XPO1-dependent export of nuclear proteins into the cytoplasm. The latter process, which we have determined is RAS dependent, takes place on the cytoplasmic face of the NPC, where the tripartite complex of Ran•GTP–XPO1–nuclear protein cargo is bound to NUP358 (ref. ^19^; Fig. 8e, step 2). Our data suggest that the RAS•GTP–RanGAP1 complex catalyzes the release of the protein cargo into the cytoplasm when Ran•GTP is hydrolyzed to Ran•GDP by RanGAP1.

Beyond establishing a mechanistically important role for the RAS•GTP–RanGAP1 complex in lung cancer, the complex appears to be a common feature of tumors with mutant RAS. It is present in PDX tumors of various origins that harbor mutant versions of KRAS, HRAS and NRAS, with less complex formation detected in tumors with wild-type RAS. This mechanism also operates in the immortalized but nontransformed HBEC cell line and in nonimmortalized WI-38 fibroblasts. However, it remains to be determined whether this is predominantly a cancer-associated RAS function or a physiologic feature of nuclear protein export. Our microscopy and subcellular fractionation studies identified the RAS•GTP–RanGAP1 complex in cytoplasmic and perinuclear locations, but not at the PM. We therefore favor a model in which the complex forms in the cytoplasm and translocates to its perinuclear location and may also form perinuclearly.

The KRAS•GTP–RanGAP1 complex has at least five notable properties. First, it places active KRAS in a perinuclear location, where we infer that it has the previously unreported activities described in this report. Therefore, this result expands the functionally important subcellular localization of active RAS^29^. Second, the KRAS•GTP–RanGAP1 complex and its downstream activities are not affected by MEKi and PI3Ki, implying that they are independent of canonical RAS signaling. Third, although both KRAS and Ran are members of the superfamily of RAS GTPases^24^, which also have their respective GAPs and GEFs, the two GTPases and their respective regulators are from different families within the superfamily. This finding represents the identification of a mechanistically important interaction between a GTPase from one family and a GAP from another family. Fourth, although the interaction between KRAS and RanGAP1 apparently facilitates Ran•GTP hydrolysis, this activity has a positive function, the cytoplasmic release of the nuclear protein cargo, thus making this a mechanistically unusual effector function for RAS, as it is mediated by its interaction with a GAP. Fifth, in addition to the noncanonical functional changes attributable to the KRAS–RanGAP1 complex, our analyses support the importance of its biological activity.

Our results have at least two potentially important clinical implications for the treatment of tumors with mutant KRAS. One is that the pro-oncogenic role we have uncovered for KRAS in nuclear protein export provides at least a partial explanation for the limited clinical success when inhibitors of canonical RAS signaling, such as MEKi and PI3Ki, have been used in cancer treatment^30,31^. The other is that, at least for lung cancers with mutant KRAS, our preclinical data have identified a feasible approach for reactivating the tumor suppressor activity of DLC1 (ref. ^4^) and suggest that it might be useful to consider combining SRCi with RASi (the AKT inhibitor might not be critical, as the RAS inhibitor would presumably blunt AKT activation).

It should be noted that Kim et al. and Khan et al. have reported a synthetic lethal interaction between mutant KRAS and XPO1 (refs. ^32,33^). However, there are numerous reports of synthetic lethal interactions with RAS that do not involve genes regulated by RAS^34^, and neither report investigated a possible regulatory relationship between KRAS and XPO1. Furthermore, Kim et al. found that XPO1i in the A549 cell line did not exhibit the synthetic lethal phenotype^32^, while this is one of the lines in which we have positive data regarding the KRAS–RanGAP1 complex and its effects on XPO1-dependent function. Mutant KRAS has also been reported to upregulate nuclear EZH2 expression in experimental pancreatic cancer and lung adenocarcinoma cell lines^35,36^.

The precise mechanism by which the KRAS–RanGAP1 complex increases the rate of XPO1-dependent export remains to be elucidated. However, our findings strongly suggest that in mutant KRAS-associated cancer, the complex substantially increases the efficiency of nuclear protein export. They also implicate farnesylation of KRAS for the most efficient binding to RanGAP1 and for stimulating XPO1-dependent export, suggesting that increased interaction with a membrane, such as the one associated with the NPC, may play a role. In this context, it may be relevant that Ran is not farnesylated^37^, in contrast to RAS and other members of the superfamily. RanGAP1 forms a dimer^38^, which makes it theoretically possible for one RanGAP1 monomer to participate in an interaction with KRAS, whereas the other RanGAP1 monomer is available for binding Ran•GTP, leading to its hydrolysis to Ran•GDP. In addition, our studies do not rule out a possible role for RAS in nuclear protein importation, which is mechanistically more heterogenous than nuclear protein export^2,3^.

## Methods

Our research complies with all relevant ethical regulations. The mouse studies were approved by the National Cancer Institute (NCI) Animal Care and Use Committee and were conducted in compliance with the approved study protocols, and the human lung cancer samples were acquired under an Emory University institutional review board-approved protocol.

### Plasmids

HA-tag vector (plasmid 38189) and HA-tagged *KRAS*^G12C^ (plasmid 58901) and *KRAS*^G12D^ (plasmid 58902) were obtained from Addgene. GFP-tagged HRAS-WT, NRAS-WT, KRAS-WT, KRAS-G12C, KRAS-G12D, KRAS-C185S, KRAS-C185S,G12C, KRAS-C185S,G12D, DDK tag vector, DDK-KRAS-WT and DDK-KRAS-K12D were provided by D. Esposito (Protein Expression Laboratory at the Frederick National Laboratory for Cancer Research).

### Antibodies and fluorescent probes

Antibody information, including the catalog numbers and dilutions, is available in the Reporting Summary linked to this article. Rabbit anti-DLC1 was generated in our laboratory (clone 428, 1:500), as described previously^39^. ‘To make the anti-DLC1 specific antibody (clone 428), the cDNA encoding a DLC1 polypeptide (amino acids 82–251) was subcloned into the bacterial expression vector PGEX-4T-1 (Pharmacia) using EcoRI and XhoI, and its encoded GST fusion protein was induced by isopropyl β-d-1-thiogalactopyranoside (IPTG) in bacteria, purified by a Glutathione Sepharose 4B column, and used to immunize rabbits’. Alexa Fluor 568 anti-rabbit IgG (1:250), Alexa Fluor 488 anti-mouse IgG (1:250) and DAPI (1:2,500) were purchased from Thermo Fisher Scientific.

### Cell lines, culture conditions and DNA transfection

NSCLC lines H1703, H157, A549, H358 and NCI-H23 were purchased from ATCC. All cancer cell lines were cultured in RPMI-1640 supplemented with 10% fetal bovine serum. HEK-293T cells and human fibroblastic WI-38 cells were purchased from ATCC and were cultured in DMEM and EMEM supplemented with 10% fetal bovine serum, respectively. HBECs were purchased from ATCC and were cultured in Airway Epithelial Cell Basal Medium with cell growth kit components. Where indicated, transient transfections were performed with Lipofectamine 3000 (Thermo Fisher Scientific) according to the manufacturer’s instructions. Stable clones expressing GFP, GFP–DLC1-WT, GFP–DLC1-K678A and GFP–DLC1-K678D were made by transfection of A549 cells with Lipofectamine 3000, followed by selection with G418 (0.9 µg ml^–1^).

### siRNA transfection and treatment of cells with serum, EGF and inhibitors

To suppress expression of specific mRNAs, cells were transfected with 160 nM siRNAs targeting *DLC1*, *XPO1*, *EZH2*, *KRAS*, *NTF2* or *RANGAP1* or with scrambled siRNAs and collected 48 h later. Suppression of protein expression, at least with two different siRNAs, was confirmed by immunoblotting. Validated siRNAs for human *DLC1* (Hs_DLC1 siRNA_5, SI03219909; Hs_DLC1 siRNA_11, SI04952213) were from Qiagen, as were scrambled siRNAs (control siRNA 1, 1027280; control siRNA 2, 1027310). The following siRNAs were purchased from Dharmacon: ON-TARGETplus Human KRAS (3845) Smart pool (L-005069-00-0005) siRNA, ON-TARGETplus Human EZH2 (2146) Smart pool L-004218-00-0005) siRNA, ON-TARGETplus Human XPO1 (7514) SMART pool (L-003030-00-0005) siRNA, ON-TARGETplus Human NUTF2 (nuclear transport factor 2) SMART pool (L-012132-00-0005) siRNA and ON-TARGETplus Human RANGAP1 SMART pool (L-006846-00-0005) siRNA. The sequences for each siRNA are described in Supplementary Table 1.

After overnight incubation in serum-free medium, cells were treated with 15% serum or EGF (purchased from Sigma-Aldrich) for 20 min. The final concentration of EGF was 100 ng ml^–1^. AKT inhibitor (mk-2206) and SRC inhibitor (saracatinib; used at 10 µM each) were purchased from Selleck Chemicals. Inhibitors for KRAS-G12C (sotorasib), KRAS-G12D (mrtx-1133), EZH2 (tazemetostat), XPO1 (selinexor), MEK (selumetinib), PI3K (copanlisib) and other pharmacological compounds (used at 1–10 µM each) were provided by the Developmental Therapeutics Program Chemicals Repository, Division of Cancer Treatment and Diagnosis, NCI.

### Coimmunoprecipitation and immunoblotting

Coimmunoprecipitation and immunoblotting were performed according to the previously described protocol^40^. ‘For co-IP experiments, equal amounts of protein from each cell lysate were precleared with Protein G Agarose (Thermo Fisher Scientific) and then incubated with the indicated antibodies or control IgG for 1 h at room temperature. After incubation, 30 µl of Protein G Agarose was added to each immune reaction and rotated overnight at 4 °C. The immunopellets were washed three times with RIPA buffer. Co-IP proteins were eluted by boiling for 5 min in 50 µl of Laemmli sample buffer containing 5% (vol/vol) 2-mercaptoethanol. Eluted proteins were resolved on a NuPage 4–12% BisTris gel and detected by IB using specific antibodies. Immunoreactive bands were detected by enhanced chemiluminescence (ECL Plus; GE Healthcare) using horseradish peroxidase-linked anti-rabbit or anti-mouse secondary antibodies’.

### Immunofluorescence staining

Immunostaining was performed according to the previously described protocol^40^. ‘Tumor tissue sections or cells were seeded onto glass chambers, incubated for 24 h, and fixed with 4% paraformaldehyde for 20 min. Fixed cells or deparaffinized tissues sections were permeabilized with 0.25% Triton X-100 in PBS and then blocked with 3% BSA in PBS for 2 h. The cells or tissue sections were incubated with the indicated primary antibodies at 4 °C overnight. After being thoroughly washed with PBS, cells were incubated with the appropriate 1:250 Alexa Fluor-conjugated secondary antibodies for 1 h. To visualize nuclei, cells were incubated with DAPI (1:2,500) for 1 h. After staining, cells were thoroughly washed with PBS and mounted with gel mounting solution (Biomeda).’

### PLA

PLA was used to visualize proximity colocalization (<40 nm) of KRAS and RanGAP1 in NSCLC lines or PDX tissue sections using a Duolink Detection kit (Olink Proteomics), as per the manufacturer’s instructions. The cells were fixed with 4% paraformaldehyde for 20 min at room temperature, and fixed cells or deparaffinized tissues sections were permeabilized with 0.25% Triton X-100 for 5 min. After incubating with Duolink blocking solution, cells were incubated overnight at 4 °C with mouse anti-KRAS (WH0003845M1; 1:200) and rabbit anti-RanGAP1 (ab92360, 1:500) or the indicated primary antibody in Duolink antibody diluent. After washing, cells were incubated with secondary antibodies conjugated to PLA probes (MINUS probe-conjugated anti-rabbit IgG and PLUS probe-conjugated anti-mouse IgG, Olink Proteomics). Circularization and ligation of the oligonucleotides in the probes were followed by an amplification step. A complementary fluorescence-labeled probe was used to detect the product of the rolling circle amplification. Slides were mounted with Duolink II mounting medium containing DAPI. Images were obtained with an LSM 780 confocal microscope (ZEISS) using ZEN software (ZEISS).

### Fluorescence confocal microscopy

Confocal microscopy was performed according to a previously described protocol^40^. ‘Confocal microscopy of fluorescent-labeled cells was performed using a confocal microscope (LSM 780; Carl Zeiss). Alexa Fluor probes were viewed with excitation wavelengths of 488 nm (Alexa Fluor 488) and 568 nm (Alexa Fluor 568). Images were made at room temperature using photomultiplier tubes with a Plan-Apochromat ×63/1.4-NA oil differential interference contrast objective lens with a 2× magnifier to produce a 125× magnification. The images were minimally processed for levels/contrast adjustment in DAPI panels, and the adjustment was done for all images using Adobe Photoshop 2024 (25.0.0) software. The colocalization of two proteins was analyzed by ZEN microscopy software (version ZEN 2.3 SP1). The adjustments do not enhance, erase or misrepresent any information present in the original images’.

### Anchorage-independent growth assay

The anchorage-independent growth assay was performed according to our previously described protocol^4^. ‘For soft agar assays, a 0.6% agar (BD) base in RPMI-1640 medium was placed in 60-mm dishes for 1 h at room temperature. 1.0 × 10^5^ cells were mixed with complete medium containing 0.4% agar and placed over 0.6% basal agar in 60-mm dishes.’ Cells were grown for 3 weeks and were continuously treated without or with selinexor, tazemetostat, sotorasib, selumetinib, copanlisib, mk-2206 and saracatinib, as indicated, and colonies were photographed microscopically and quantified with a colony counter.

### Generation of *DLC1*-KO NCI-H23 cells

CRISPR–Cas9-mediated knockout of *DLC1* in the NCI-H23 lung cancer cell line was performed by targeting exon 5 of *DLC1* transcript variant 2 (ref. ^4^; NM_006094). The targeted region of *DLC1* was amplified from NCI-H23 clones that were negative for DLC1 protein expression by western blotting, and PCR products were sequenced to confirm the presence of insertion/deletion mutations that would cause premature translation termination of the DLC1 polypeptide. Cells were transfected with two different constructs (pAG0266 and pAG0267) with single-guide RNA (sgRNA) for *DLC1*. Lenti-SpCas9-2A-GFP-DLC1-IVT vector was used to deliver individual sgRNAs. The sequences of sgRNA primers for *DLC1* and nontargeted control sgRNA are described in Supplementary Table 2. Lipofectamine 3000 (Life Technologies) was used to transfect plasmid DNA according to the manufacturer’s instructions. GFP^+^ single cells were sorted using a FACSAria UV into a sterile 96-well culture plate, yielding single-cell *DLC1*-KO clones.

### Purification of recombinant proteins and preparation of exclusively GDP-bound and GTP analog GppNHp-bound KRAS proteins

DDK–RanGAP1 (Origene) and DDK–CDCP1 (a gift from the laboratory of B. Mock at the NCI) were transfected into HEK-293T cells for 48 h and lysed, as described previously^41^. ‘Two days after transfection, cells were lysed with Golden Lysis Buffer (GLB: 20 mM Tris (pH 7.9), 137 mM NaCl, 10% glycerol, 1% Triton, 5 mM EDTA, 1 mM EGTA, 1 mM Na_3_VO_4_, 10 mM NaF, 1 mM sodium pyrophosphate, 0.5 mM β-glycerophosphate and protease inhibitor cocktail tablet (Roche)). The cleared supernatants were collected, and the amount of protein estimated by BCA kit (Pierce).’ The cell extracts were immunoprecipitated by DDK Flag beads (Sigma-Aldrich) and washed thoroughly with HNTG (20 mM HEPES buffer (pH 7.5) containing 150 mM NaCl, 0.1% (wt/vol) Triton X-100 and 10% (wt/vol) glycerol) buffer. The purified GST-tagged RAF-RBD protein was purchased from EMD Millipore. KRAS4b (1–169) expression clones of both wild-type and KRAS-G12D mutant for *Escherichia coli* production were generated using a His×6–maltose-binding protein fusion. All proteins were purified as outlined for G-Hs.KRAS4b (1–169), as described previously^42^. ‘Cell pellets were resuspended in 20 mM HEPES, pH 7.3, 300 mM NaCl, 1 mM TCEP and 1:200 (vol/vol) protease inhibitor cocktail. Homogenized cells were lysed by passing twice through a Microfluidizer at 9,000 psi. Lysates were clarified by centrifugation at 7,900*g* for 90 min at 4 °C. Clarified lysates were filtered through 0.45-μm Whatman PES syringe filters, and proteins were purified using NGC medium-pressure chromatography systems. Clarified lysates were thawed, adjusted to 35 mM imidazole and loaded at 3 ml min^–1^ onto IMAC columns equilibrated in IMAC equilibration buffer (EB) of 20 mM HEPES, pH 7.3, 300 mM NaCl, 1 mM TCEP, 35 mM imidazole and 1:1,000 protease inhibitor cocktail. The columns were washed to baseline with EB and proteins eluted with a 20 column-volume gradient from 35 mM to 500 mM imidazole in EB. Elution fractions were analyzed by SDS–PAGE.’ The quantity of all purified proteins was estimated by Coomassie blue stained gel compared to known concentrations of the albumin standard. KRAS nucleotide exchange efficiency was determined using high-performance liquid chromatography. Exchanged proteins were diluted into 0.1 M K_2_HPO_4_ and 1 mM tetrabutyl ammonium hydrogen sulfate (buffer A) and injected onto an Ultrasphere 5 ODS, 250 ×4.6 mm column (Hichrom). Bound nucleotides were eluted using a linear gradient of buffer A containing 30% acetonitrile at a flow rate of 0.6 ml min^–1^. Standards of GDP and GMPPNP (GTP) were included to validate the identity of the bound nucleotide. GMPPNP exchange efficiency was routinely >95% pure as measured by this assay.

### Generation of full-length GST–RanGAP1 and truncated (1–416) GST–RanGAP1 constructs by PCR cloning

DDK–RanGAP1 expressing wild-type RanGAP1 was used as a template. The designed PCR primers included 5′ KpnI and 3′ NotI restriction sites. All primer sequences are described in Supplementary Table 2. Twenty cycles of PCR were performed. The PEBG mammalian expression vector^41^ was used for GST-tagged proteins after subcloning the PCR products with KpnI and NotI restriction sites.

### In vitro KRAS–RanGAP1 binding assay

Purified KRAS proteins (wild-type KRAS or KRAS-G12D) were mixed with purified DDK–RanGAP1, DDK–CDCP1 or RAF-RBD in Mg^++^ lysis buffer (EMD Millipore) and rotated for 3 h at 4 °C, followed by washing with HNTC buffer. The pulldown beads were resuspended in 50 μl of Laemmli sample buffer separated on a reducing SDS–PAGE gel and immunoblotted with antibodies to DDK and KRAS, followed by secondary anti-IgG conjugated to anti-horseradish peroxidase (1:5,000). The signals bound to the membranes were detected by an ECL plus kit (GE Healthcare).

### Nuclear and cytoplasmic fractionation

Nuclear and cytoplasmic fractionation was performed according to our previously described protocol^4^. ‘Nuclear and cytoplasmic fractions of cells were purified using a Nuclear/Cytosolic Fractionation Kit (AKR-171, Cell Biolabs), as per the manufacturer’s instructions. Briefly, cells were pelleted by centrifugation for 5 min at 4 °C (600*g*) and washed with ice-cold PBS. The cell pellets were resuspended with 500 μl of ice-cold extraction buffer containing DTT and protease inhibitors. The cell suspension was transferred into a prechilled microcentrifuge tube and incubated on ice for 10 min, 25 μl of cell lysis reagent was added, vortexed for 10 s and centrifuged at 800*g* for 10 min at 4 °C. The resulting supernatant (cytoplasmic fraction) was transferred to a clean, chilled microcentrifuge tube and stored at –80 °C until use. For nuclear protein extraction, the pellet was gently resuspended in 100 μl of ice-cold nuclear extraction buffer containing DTT and protease inhibitors, incubated on ice for 30 min, vortexed for 10 s and centrifuged at 14,000*g* for 30 min at 4 °C. The supernatant (nuclear protein extract) was stored at –80 °C until use. All buffers were supplemented with protease cocktail and phosphatase inhibitors’.

### PM isolation

The PM was isolated using a Minute Plasma Membrane Protein Isolation and Cell Fractionation kit (SM-005, Invent Biotechnologies), as per the manufacturer’s instructions. Cells were pelleted by centrifugation for 5 min at 4 °C (600*g*), washed with cold PBS, incubated with Buffer A for 10 min on ice, vortexed at high speed for 30 s, transferred to a prechilled filter cartridge assembly collection tube and centrifuged at 16,000*g* for 30 s at 4 °C. The pellet was resuspended and centrifuged at 700*g* for 1 min, and the supernatant was transferred to a new microcentrifuge tube and centrifuged at 16,000*g* for 30 min at 4 °C. For the PM, the pellet was resuspended in 200 μl of Buffer B by vortexing at moderate intensity for 30 s and centrifuging at 7,800*g* for 5 min at 4 °C. The supernatant was transferred to a new microcentrifuge tube, and 1.5 ml of chilled PBS was added, mixed vigorously for 30 s and centrifuged at 16,000*g* for 30 min at 4 °C. The pellet containing the PM was resuspended in RIPA buffer with protease and phosphatase inhibitors plus 1.0% Triton X-100.

### NE isolation

The NE was isolated using a Minute Nuclear Envelope Protein Extraction kit (NE-013, Invent Biotechnologies), as per the manufacturer’s instructions. Cells were pelleted by centrifugation at 600*g* for 5 min at 4 °C, washed twice with PBS, resuspended in Buffer A, incubated for 10 min on ice, mixed vigorously, transferred into a prechilled filter cartridge assembly tube and centrifuged at 14,000*g* for 30 s at 4 °C. The pellet was washed with cold PBS, resuspended in Buffer B by vortexing, incubated on ice for 5 min and centrifuged at 5,000*g* for 5 min at 4 °C. The supernatant was transferred to a new tube, and 1.0 ml of cold PBS was added, mixed vigorously for 15 s and centrifuged at 16,000*g* for 15 min at 4 °C. The NE pellet was then resuspended in RIPA buffer with protease and phosphatase inhibitors plus 0.25% Triton X-100.

### Ran•GTP assay

A Ran activation assay kit (81701, NewEast Biosciences) was used for measuring GTP-bound Ran, as per the manufacturer’s instructions. Briefly, equal amounts of each cytoplasmic fraction (1,000 µg of protein) was incubated with 2 µl of anti-Ran•GTP for 1 h. After incubation, 30 µl of Protein A/G Agarose was added to each immune reaction and rotated at 4 °C for 2 h. The beads were then washed three times with washing buffer. Washed samples were subjected to separation on 4–12% SDS–PAGE gels, transferred onto nitrocellulose membranes and detected by immunoblotting using antibody to Ran (Cell Signaling Technology).

### Primary human lung adenocarcinoma samples

The primary human lung adenocarcinoma samples were provided by the lung SPORE from Winship Cancer Institute, Emory University. Snap-frozen remnant lung tumor tissues were obtained from deidentified individuals by assigning random IDs for the purpose of this study under an Emory University institutional review board-approved protocol.

### PDX models

Flash-frozen tumor fragments from PDX models 941728-121-R (lung adenocarcinoma), 422866-222-R5 (pancreatic adenocarcinoma), 463931-005-R (pancreatic adenocarcinoma), 572918-348-R (colon adenocarcinoma), 144555-231-T (colon adenocarcinoma), 765993-094-R (nasopharyngeal carcinoma with mutant HRAS-G12D) and 782815-120-R (colon adenocarcinoma with mutant NRAS-Q61R) were obtained from the NCI’s Patient Derived Models Repository (NCI Frederick, Frederick National Laboratory for Cancer Research; https://pdmr.cancer.gov/). Flash-frozen tumor fragments from PDX models LG0703-F948 (lung adenocarcinoma) and K00052-001-T (lung adenocarcinoma) were developed by The Jackson Laboratory and are available from the NCI Patient Derived Models Repository.

### Development and treatment of the KRAS-G12D mouse lung cancer model

All mouse studies were approved by the NCI Animal Care and Use Committee and were conducted in compliance with the approved protocols. The animals were housed under standard laboratory conditions on a 12-h dark/12-h light cycle (0600 to 1800 h) at ambient temperature 20–24 °C with 30–70% humidity and were provided with continuous food and water. Mouse lung tumors were generated by conditional expression of oncogenic KRAS and inactivation of p53 (ref. ^25^). The *Kras*^LSL-G12D/+^ (B6.129S4-*Kras*^*tm4Tyj*^/J) and *Trp53*^fl/fl^ (B6.129P2-*Trp53*^*tm1Brn*^/J) mouse strains were purchased from The Jackson Laboratory and were bred to produce *Kras*^LSL-G12D/+^; *Trp53*^fl/fl^ mice. Adenovirus expressing Cre recombinase (Ad5CMVCre) was provided by the University of Iowa Viral Vector Core Facility, and a dose of 2.5 × 10^7^ plaque-forming units per mouse was delivered to the respiratory tract of mice anesthetized with isoflurane, using the modified intranasal method of Santry et al.^43^. Three months after adenovirus infection, mice were randomly divided into groups and were treated daily for 3 weeks via intraperitoneal injection of KRAS-G12D inhibitor mrtx-1133 (15 mg per kg (body weight)) alone, oral administration of the XPO1 inhibitor selinexor (30 mg per kg (body weight)) alone, the three-drug combination of mrtx-1133 (15 mg per kg (body weight)) + saracatinib (50 mg per kg (body weight)) + mk-2206 (50 mg per kg (body weight)) or selinexor (30 mg per kg (body weight)) + saracatinib (50 mg per kg (body weight)) + mk-2206 (50 mg per kg (body weight)), the two-drug combination of selumetinib (15 mg per kg (body weight)) + copanlisib (15 mg per kg (body weight)) or saracatinib (50 mg per kg (body weight)) + mk-2206 (50 mg per kg (body weight)) or vehicle (Captisol), and intact lungs with residual tumors were then excised and processed for biochemical assays after treatment.

### In vivo tumorigenesis and treatment of mice with inhibitors

For the development and treatment of mice with xenograft tumors, A549 or NCI-H23 cells with *DLC1*-WT or *DLC1*-KO were trypsinized, washed with cold PBS, diluted to 10^7^ cells per ml with serum-free medium/Matrigel basement membrane matrix (BD Biosciences) at a ratio of 3:1 and injected subcutaneously into NOD-*scid* mice (10^6^ cells per injection). When tumors were approximately 0.5 cm in diameter, mice were randomly divided into groups and were treated daily with oral EZH2 inhibitor tazemetostat (25 mg per kg (body weight)), the XPO1 inhibitor selinexor (30 mg per kg (body weight)) or the KRAS-G12C inhibitor sotorasib (15 mg per kg (body weight)), the two-drug combination of selumetinib (15 mg per kg (body weight)) + copanlisib (15 mg per kg (body weight)) for 1 week, followed by treatment with the indicated combination of tazemetostat (25 mg per kg (body weight)) + saracatinib (50 mg per kg (body weight)) + mk-2206 (50 mg per kg (body weight)) or sotorasib (15 mg per kg (body weight)) + saracatinib (50 mg per kg (body weight)) + mk-2206 (50 mg per kg (body weight)), all three drugs in the indicated combination or vehicle (Captisol) for an additional 2 weeks, and the remaining tumor tissues were excised, weighed and processed for biochemical assays after treatment. The maximal tumor size was not exceeded as permitted by the ethics committee and approved protocols. Sex was not considered in the study design because sex-based analysis was not relevant to the study. Therefore, this information was not collected.

### Data analysis, statistics and reproducibility

At least two independent experiments were performed for all experiments. Immunoblots were quantified by densitometric scanning using Fiji software. Results in bar graphs are displayed as mean values ± s.d. from two or three experiments. No statistical methods were used to predetermine sample sizes, but our sample sizes are similar to those reported in previous publications within this field of research^4,28^. All animal experiments were grouped randomly based on genetically related cohorts and tumor size. For all other experiments, the sample allocation was random. The investigators were blinded to group allocation during data collection and/or analysis. No animals and data points were excluded from the analyses. All experiments were designed with matched control conditions within each experiment. Data distribution was assumed to be normal, but this was not formally tested. For the statistical analyses, parametric unpaired one-tailed *t-*test with Welch’s correction was performed using Prism software (version 10.1.1 (270), GraphPad), and no adjustments were made for multiple comparisons. A *P* value of <0.05 was considered statistically significant.

### Reporting summary

Further information on research design is available in the Nature Portfolio Reporting Summary linked to this article.

## Supplementary information

Reporting Summary

Supplementary Table 1 Supplementary Table 1. Sequences of all siRNAs used in this study. Supplementary Table 2. Sequences for all sgRNAs and primers used in this study.

## Source data

Source Data Fig. 3 Statistical source data.

Source Data Fig. 7 Statistical source data.

Source Data Fig. 8 Statistical source data.

Source Data Extended Data Fig. 7 Statistical source data.

Source Data Extended Data Fig. 8 Statistical source data.

Source Data Figs. 1–3, 5 and 6 and Extended Data Figs. 2, 3 and 6 Unprocessed and uncropped western blots and gels.

## Data Availability

All data generated or analyzed during this study are included in the published article and its Supplementary Information files. Source data are provided with this paper.

## Code availability

No new code was developed in this study.
