# Supplementary material for: The pro-oncogenic noncanonical activity of a RAS•GTP:RanGAP1 complex facilitates nuclear protein export
Source: Nat Cancer. 2024 Nov 11;5(12):1902–18. doi: 10.1038/s43018-024-00847-5 (PMC11663792; doi:10.1038/s43018-024-00847-5)
Supplement: Supplementary file 1 — Reporting Summary [file 43018_2024_847_MOESM1_ESM.pdf]

nature portfolio

Corresponding author(s): Dr. Douglas R. Lowy; Dr. Brajendra K. Tripathi

Last updated by author(s): Sep 18, 2024

Reporting Summary

Nature Portfolio wishes to improve the reproducibility of the work that we publish. This form provides structure for consistency and transparency in reporting. For further information on Nature Portfolio policies, see our Editorial Policies and the Editorial Policy Checklist.

Statistics

For all statistical analyses, confirm that the following items are present in the figure legend, table legend, main text, or Methods section.

n/a	Confirmed	
☐	☒	The exact sample size (*n*) for each experimental group/condition, given as a discrete number and unit of measurement
☐	☒	A statement on whether measurements were taken from distinct samples or whether the same sample was measured repeatedly
☐	☒	The statistical test(s) used AND whether they are one- or two-sided<br>*Only common tests should be described solely by name; describe more complex techniques in the Methods section.*
☒	☐	A description of all covariates tested
☒	☐	A description of any assumptions or corrections, such as tests of normality and adjustment for multiple comparisons
☐	☒	A full description of the statistical parameters including central tendency (e.g. means) or other basic estimates (e.g. regression coefficient) AND variation (e.g. standard deviation) or associated estimates of uncertainty (e.g. confidence intervals)
☐	☒	For null hypothesis testing, the test statistic (e.g. *F*, *t*, *r*) with confidence intervals, effect sizes, degrees of freedom and *P* value noted<br>*Give P values as exact values whenever suitable.*
☒	☐	For Bayesian analysis, information on the choice of priors and Markov chain Monte Carlo settings
☒	☐	For hierarchical and complex designs, identification of the appropriate level for tests and full reporting of outcomes
☒	☐	Estimates of effect sizes (e.g. Cohen's *d*, Pearson's *r*), indicating how they were calculated

Our web collection on statistics for biologists contains articles on many of the points above.

Software and code

Policy information about availability of computer code

Data collection	Confocal microscopy of fluorescent-labeled cells was performed using a confocal microscope (LSM 780; Carl Zeiss). Alexa Fluor probes were viewed with excitation wavelengths of 488 nm (Alexa Fluor 488) and 568 nm (Alexa Fluor 568). Images were made at RT using photomultiplier tubes with a Plan-Apochromat 63×/1.4 NA oil differential interference contrast objective lens.
Data analysis	The colocalization of two proteins was analyzed by ZEN microscopy software (version ZEN 2.3 SP1). For the statistical analyses, parametric unpaired one tailed t-test with Welch's correction was performed using Prism software (version 10.1.1 (270); GraphPad) and no adjustments were made for multiple comparisons, and p < 0.05 was considered statistically significant.

For manuscripts utilizing custom algorithms or software that are central to the research but not yet described in published literature, software must be made available to editors and reviewers. We strongly encourage code deposition in a community repository (e.g. GitHub). See the Nature Portfolio guidelines for submitting code & software for further information.

Data

Policy information about availability of data

All manuscripts must include a data availability statement. This statement should provide the following information, where applicable:

- Accession codes, unique identifiers, or web links for publicly available datasets
- A description of any restrictions on data availability
- For clinical datasets or third party data, please ensure that the statement adheres to our policy

All data generated or analyzed during this study are included in the article and its supplementary information files. All the data are available within the article and its supplementary information. Source Data are provided with this paper.

Research involving human participants, their data, or biological material

Policy information about studies with human participants or human data. See also policy information about sex, gender (identity/presentation), and sexual orientation and race, ethnicity and racism.

Reporting on sex and gender	There were no sex or gender based analysis in this study, and this information has not been collected. Sex and gender were not considered in the design of the study.
Reporting on race, ethnicity, or other socially relevant groupings	This information was not provided and was not considered in the design of the study.
Population characteristics	No data on population characteristics was collected/used.
Recruitment	The recruitment of the patients were done as per an Emory University institutional review board approved protocol. For the purpose of this study, lung tumor tissues were obtained from the de-identified patients by assigning random IDs.
Ethics oversight	The primary human lung adenocarcinoma samples were provided by the lung SPORE from Winship Cancer Institute, Emory University, Atlanta, GA USA. Snap-frozen, remnant lung tumor tissues were obtained from the de-identified patients by assigning random IDs for the purpose of this study under an Emory University institutional review board approved protocol. The written consent was obtained from the patients the patient samples derive from, as per requirements for the Emory University institutional review board approved protocol.

Note that full information on the approval of the study protocol must also be provided in the manuscript.

Field-specific reporting

Please select the one below that is the best fit for your research. If you are not sure, read the appropriate sections before making your selection.

☒ Life sciences ☐ Behavioural & social sciences ☐ Ecological, evolutionary & environmental sciences

For a reference copy of the document with all sections, see nature.com/documents/nr-reporting-summary-flat.pdf

Life sciences study design

All studies must disclose on these points even when the disclosure is negative.

Sample size	At least two independent experiments were performed for all in vitro experiments and at least four animals (n=4) were used per group for all in vivo experiments. Sample sizes were determined based on our experience with the specific type of experiment and commonly used sample sizes in ours and others previous publications within this field of research (Tripathi et al., 2017, Journal of Cell Biology; Tripathi et al., 2019, Journal of Cell Biology, Tripathi et al., Nature Communications, 2021). The sample sizes and number of repeats are also defined in each figure legends. Immunoblots were quantified by densitometric scanning using Fiji software 2.14.0. Results are expressed as mean ± standard deviation (SD) from two or three experiments. All experiments were designed with matched control conditions within each experiment. For the statistical analyses, parametric unpaired one tailed t-test with Welch's correction was performed using Prism software (version 10.1.1 (270); GraphPad) and no adjustments were made for multiple comparisons, and $p < 0.05$ was considered statistically significant.
Data exclusions	No data were excluded from the analysis.
Replication	At least two independent experiments were performed for all experiments. All attempt at replication were successful. At least four animals (n=4) were used per group for all in vivo experiments. The experimental findings reported in this article were reliably reproduced. All other experiments were performed independently two times and all replication attempts were successful. Results in bar graphs are displayed as mean ± standard deviation (SD) from two or three experiments. All experiments were designed with matched control conditions.
Randomization	All animal experiments were grouped randomly based on genetically related cohorts and tumor size. When tumors were approximately 0.5 cm in diameter, mice were randomly divided into groups and were treated with drugs. For all other experiments, the sample allocation was random and the investigators were blinded to group allocation.

Blinding	The investigators were blinded to group allocation during data collection and/or analysis.

Reporting for specific materials, systems and methods

We require information from authors about some types of materials, experimental systems and methods used in many studies. Here, indicate whether each material, system or method listed is relevant to your study. If you are not sure if a list item applies to your research, read the appropriate section before selecting a response.

Materials & experimental systems

n/a	Involved in the study	
[ ]	[x]	Antibodies
[ ]	[x]	Eukaryotic cell lines
[x]	[ ]	Palaeontology and archaeology
[ ]	[x]	Animals and other organisms
[x]	[ ]	Clinical data
[x]	[ ]	Dual use research of concern
[x]	[ ]	Plants

Methods

n/a	Involved in the study	
[x]	[ ]	ChIP-seq
[x]	[ ]	Flow cytometry
[x]	[ ]	MRI-based neuroimaging

Antibodies

Antibodies used	The following antibodies, with the catalog number and the dilution used in parenthesis, were purchased from Cell Signaling Technology: XPO1 (46249, 1:1000), ERK (9102, 1:1000), pERK-Thr202/Tyr204 (9101, 1:1000), AKT (4691, 1:1000), phospho-AKT-pS473 (4060, 1:1000), SRC (2108, 1:1000), phospho-SRC-pY416 (2101, 1:1000), EZH2 Rabbit (5246, 1:1000), EZH2 Mouse (3147, 1:1000) Survivin Rabbit (2808, 1:1000), Survivin Mouse (2802, 1:1000), NTF2 (3053, 1:1000), RAN (4462, 1:1000), CDC42 (2466, 1:1000), GST Mouse (2624, 1:1000) GST Rabbit (2622, 1:2000), FAK (3285, 1:1000), CD44 (37259, 1:1000), EGFR (4267, 1:1000), Lamin A/C (4777, 1:2000), and GAPDH (2118, 1:5000). RAN-GAP (67146, 1:1000) and KRAS (12063-1-AP, 1:1000) were purchased from Proteintech. RAN-GAP Mouse (33-0800, 1:1000) and KRAS Mouse (415700, 1:500) were purchased from Invitrogen. KRAS-G12D (26036, 1:500), RAN (21097, 1:1000) and RAN-GTP (26915, 1:500) were purchased from NewEast Biosciences. DDK Mouse (TA50011, 1:2000) was purchased from Origene. Two DLC1 antibodies were used: one, generated in our laboratory (DLC1 Rabbit antibody; clone 428, 1:500), and the other DLC1 Mouse (612021, 1:500) was purchased from BD Biosciences. RAS (ab180772, 1:1000), KRAS (ab275876, 1:1000), [illegible]-Tubulin (ab4074, 1:5000), Lamin B1 (ab65986, 1:500), NTF2 (ab254146, 1:1000), KRAS-G12D (ab221163, 1:500), BRAF (ab33899, 1:1000), RANBP1 (ab97659, 1:500), RANBP2 (NUP358, ab245563, 1:500), RAP1 Mouse (ab175329, 1:500), RAP1 Rabbit (ab272863, 1:500), RAP1-GAP (ab32373, 1:500), GFP mouse (ab1218, 1:1000), GFP Rabbit (ab290, 1:2000), and RAN-GAP (ab92360, 1:1000) antibodies were purchased from Abcam. RAP1 (07-196, 1:500) antibody was purchased from EMD Millipore. NUP358 (RANBP2; sc74518, 1:500) and DLC1 (271915, 1:200) antibody was purchased from Santa Cruz Biotechnology. KRAS mouse (WH0003845M1, 1:200), Vinculin (V9131, 1:1000), and Actin (A4700, 1:3000) antibodies were purchased from Sigma Aldrich. Anti-Rabbit (NA934V, 1:5000) and anti-Mouse (NXA931V, 1:3000) IgG horseradish peroxidase-linked secondary antibodies were purchased from GE Healthcare.
Validation	We have used and validated most of the antibodies that are listed here in our previous studies (Tripathi et al., Journal of Cell Biology, 2014; Tripathi et al., Journal of Cell Biology, 2017; Tripathi et al., Journal of Cell Biology, 2019, Tripathi et al., Nature Communications, 2021). For validation of new antibodies, we have used antibody profiles of online databases from the company site and the relevant citation for each primary antibody. All new antibodies used in this study were procured directly from manufacturers and were validated by the manufacturers for both antigen specificity and reactivity with human cells using western blot, immunoprecipitation and/ or immunoblotting. Comprehensive details on the validation procedures for each antibody are available in the technical datasheet and references provided by the respective manufacturers. All antibodies used in this study exhibited expected immunoblotting and immunostaining results in accordance with the existing literature.

Eukaryotic cell lines

Policy information about cell lines and Sex and Gender in Research

Cell line source(s)	HEK-293T cells and human fibroblastic WI-38 cells were purchased from ATCC and were cultured in DMEM and EMEM supplemented with 10% FBS, respectively. Human Bronchial Epithelial cells (HBEC) were purchased from ATCC and were cultured in Airway Epithelial Cell Basal Medium with cell growth kit components. NSCLC lines H1703, H157, A549, NCI-H23, and H358 were purchased from ATCC. All cancer cell lines were cultured in RPMI-1640 supplemented with 10% FBS. All cells were cultured at 37°C in a humidified atmosphere of 95% air and 5% CO_2.
Authentication	We have used the reliable sources for each cell lines, such as ATCC, but we did not authenticated. STR analysis and COI assay were used to authenticate each cell line by the supplier.
Mycoplasma contamination	All cell lines tested negative for mycoplasma contamination.
Commonly misidentified lines (See ICLAC register)	No misidentified cell lines have been used in this study.

Animals and other research organisms

Policy information about studies involving animals; ARRIVE guidelines recommended for reporting animal research, and Sex and Gender in Research

Laboratory animals	The mouse studies were approved by the National Cancer Institute Animal Care and Use Committee and conducted in compliance with the approved protocols. NOD.SCID/NCR mice were obtained from Charles River Laboratory, National Cancer Institute centralized animal order system. We used 6-8 weeks old mice for this study. The animals were housed under standard laboratory conditions in 12 hours dark/light cycle (6 am to 6 pm) at ambient temperature 68-76F with 30%-70% humidity and were provided continuous food and water supply. Mouse lung tumors were generated by conditional expression of oncogenic Kras and inactivation of p53. The KrasLSL-G12D/+ (B6.129S4-Krastm4Tyj/J) and Trp53fl/fl (B6.129P2-Trp53tm1Brn/J) mouse strains were purchased from The Jackson Laboratories and were bred to produce KrasLSL-G12D/+; Trp53fl/fl mice. Adenovirus expressing Cre recombinase (Ad5CMVCre) was provided by the University of Iowa Viral Vector Core Facility, and a dose of 2.5x107 pfu per mouse was delivered to the respiratory tract of mice anesthetized with isoflurane. The maximal tumor size was not exceeded to the permitted by the ethics committee and approved protocols.
Wild animals	The study did not involve wild animals.
Reporting on sex	Sex and gender based analysis were not relevant to the study, and this information has not been collected. Sex and gender were not considered in the study design.
Field-collected samples	No field collected samples were used in the study
Ethics oversight	The mouse studies were approved by the National Cancer Institute Animal Care and Use Committee and conducted in compliance with the approved protocols. Animals were housed under standard laboratory conditions and water and food were continuously available.

Note that full information on the approval of the study protocol must also be provided in the manuscript.
