# Supplementary material for: The pro-oncogenic noncanonical activity of a RAS•GTP:RanGAP1 complex facilitates nuclear protein export
Source: Nat Cancer. 2024 Nov 11;5(12):1902–18. doi: 10.1038/s43018-024-00847-5 (PMC11663792; doi:10.1038/s43018-024-00847-5)

Uncropped blots for Figure 1

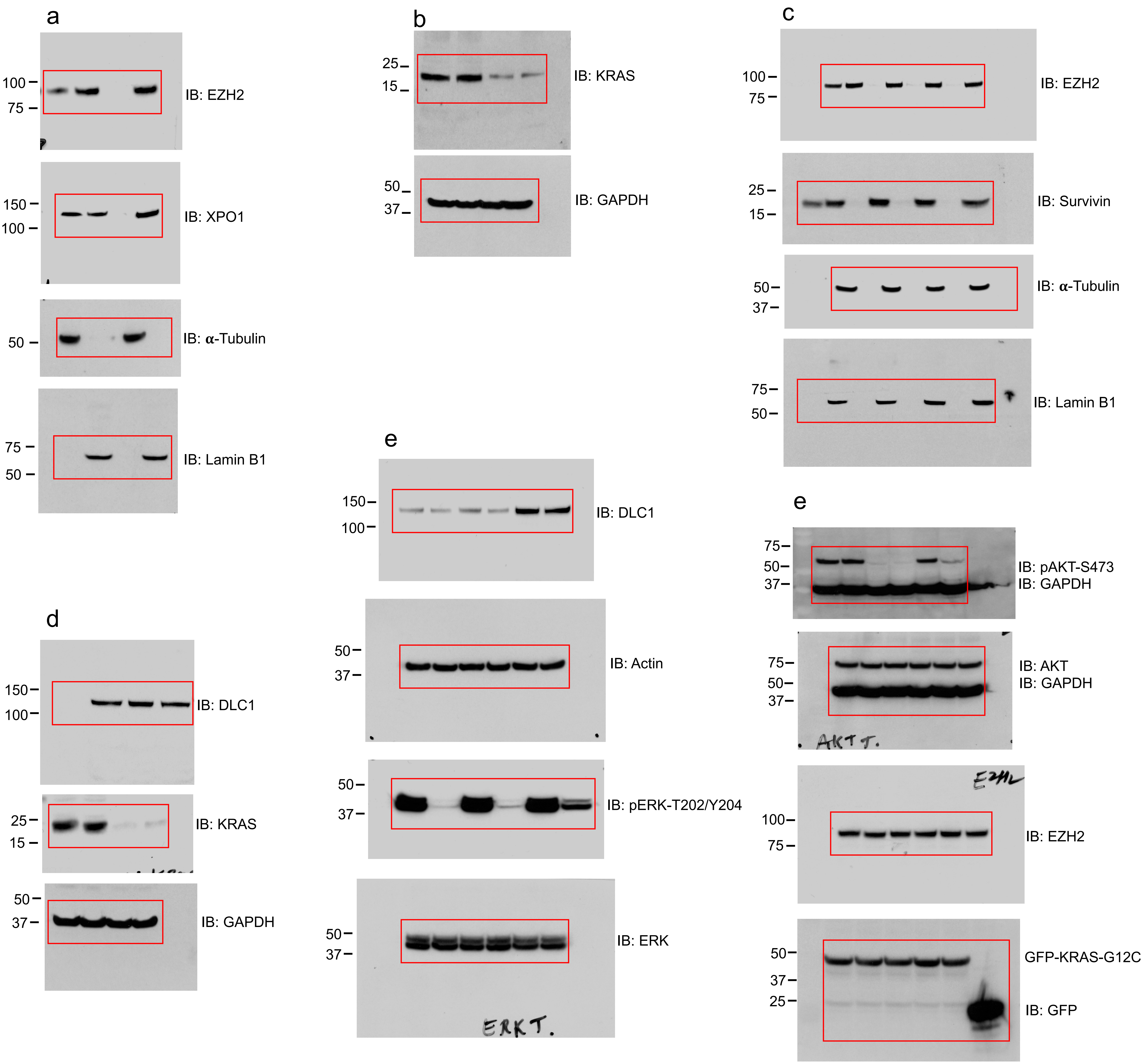

Uncropped blots for Figure 1 continue

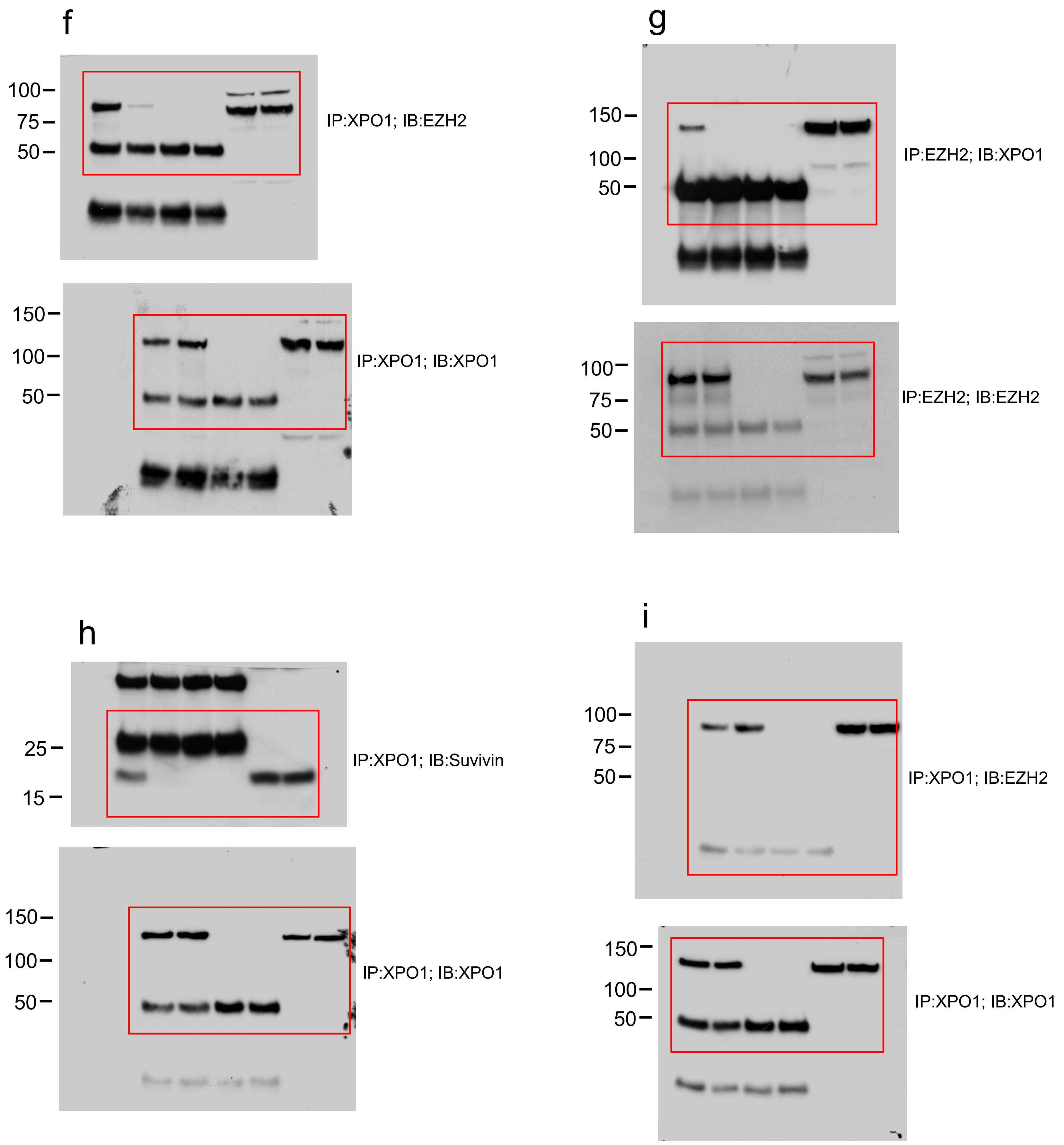

Uncropped blots for Figure 2

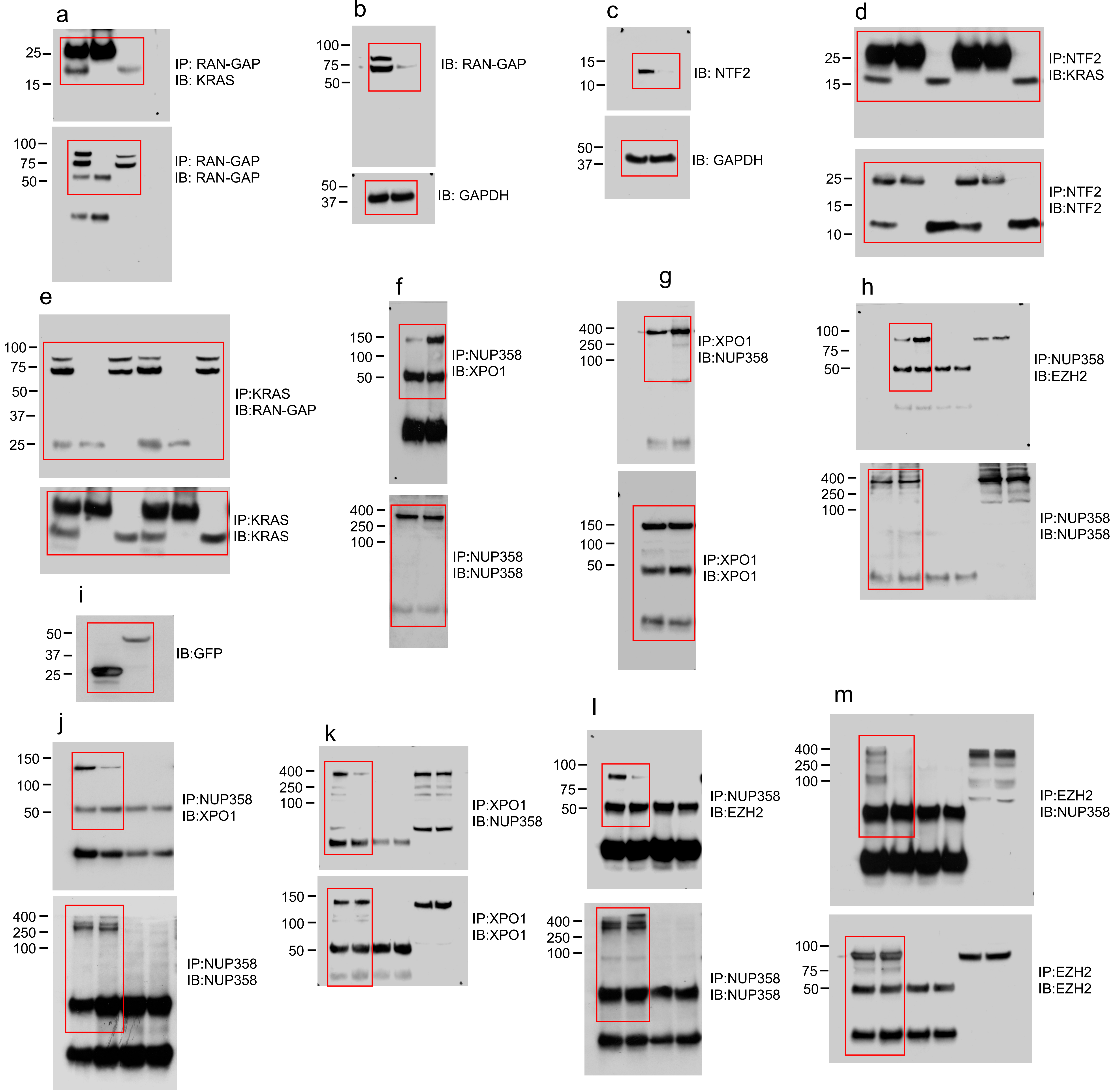

Uncropped blots for Figure 3

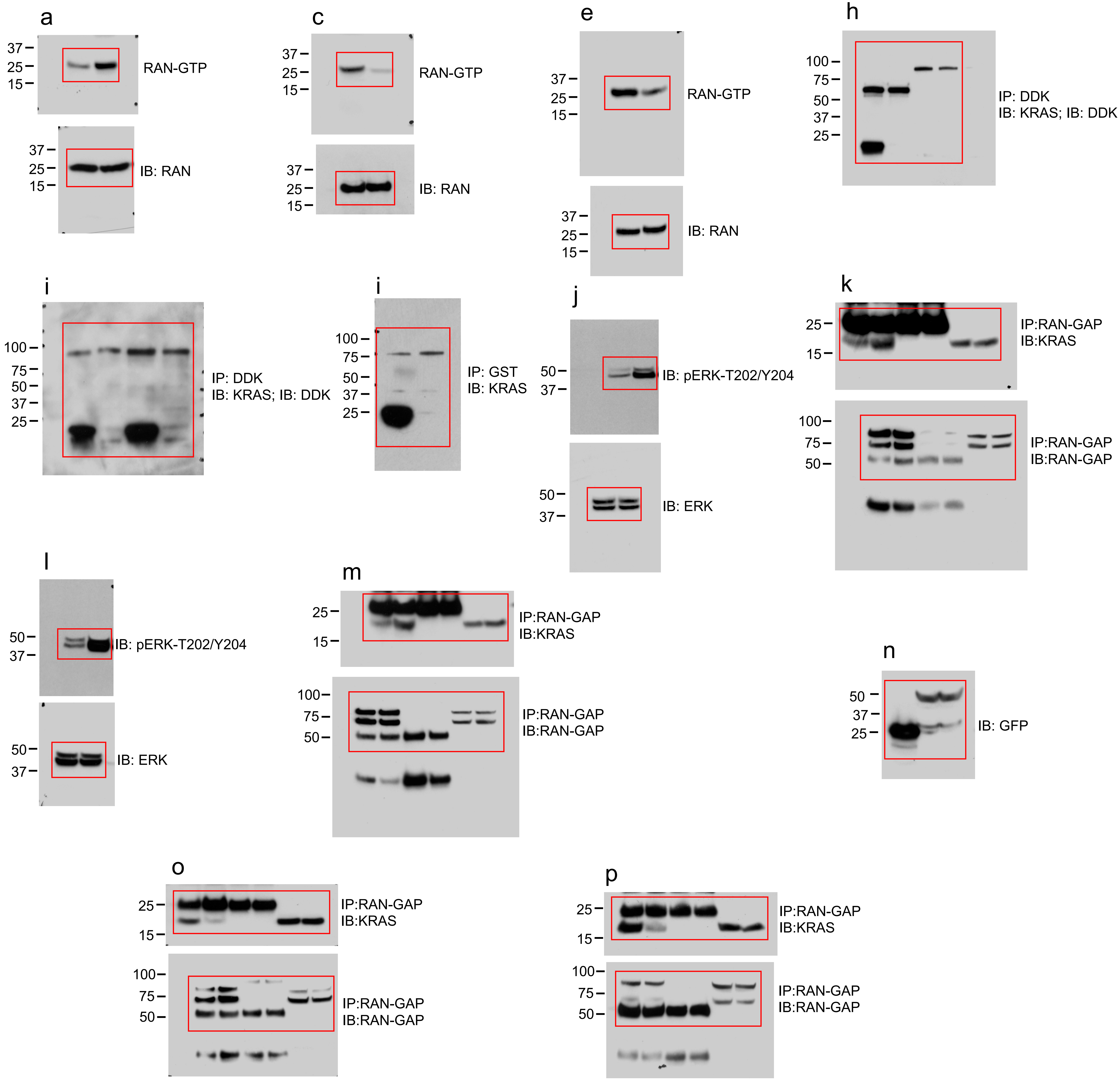

Uncropped blots for Figure 5

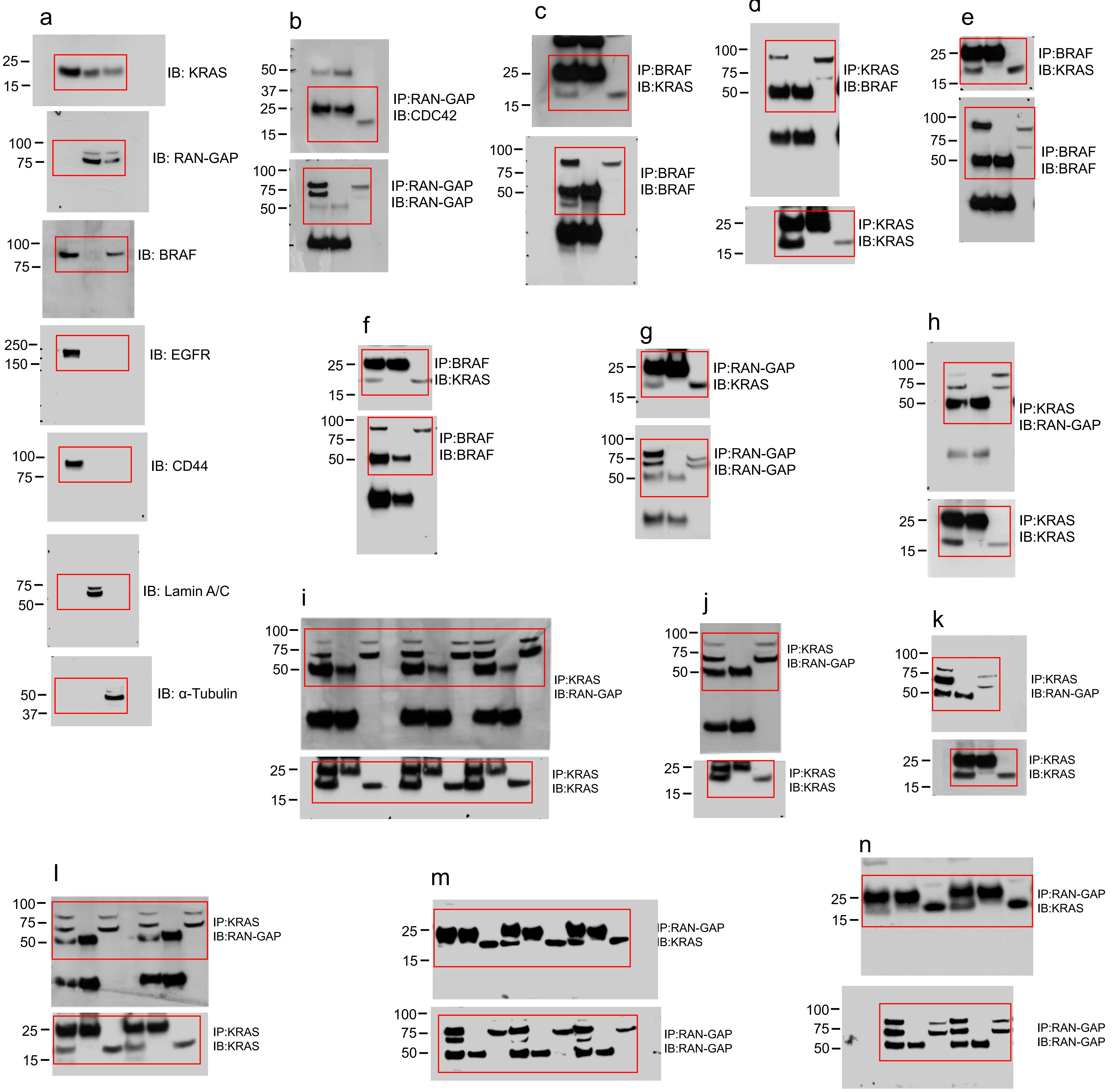

Uncropped blots for Figure 6

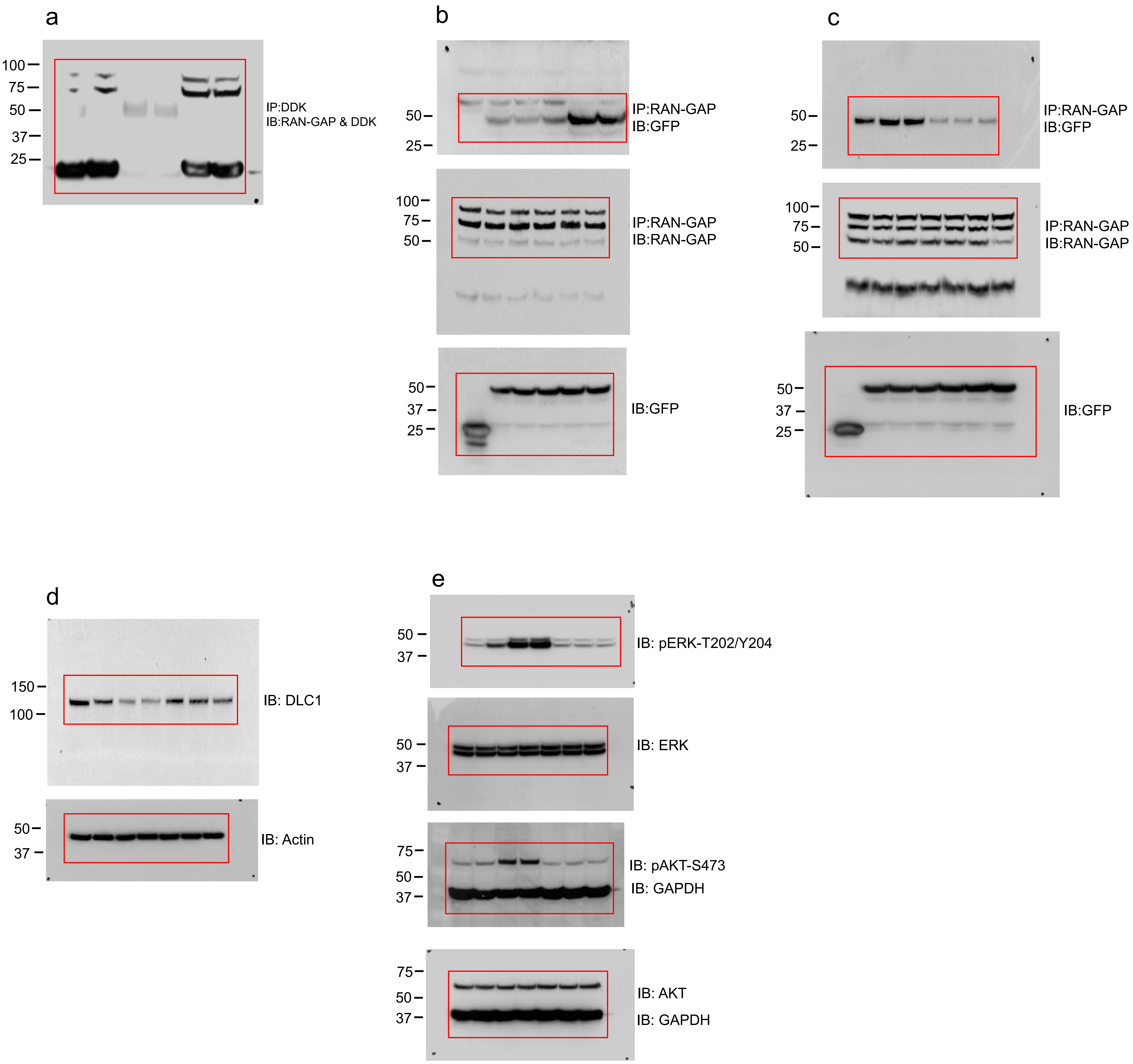

Uncropped blots for Extended Data Figure 2

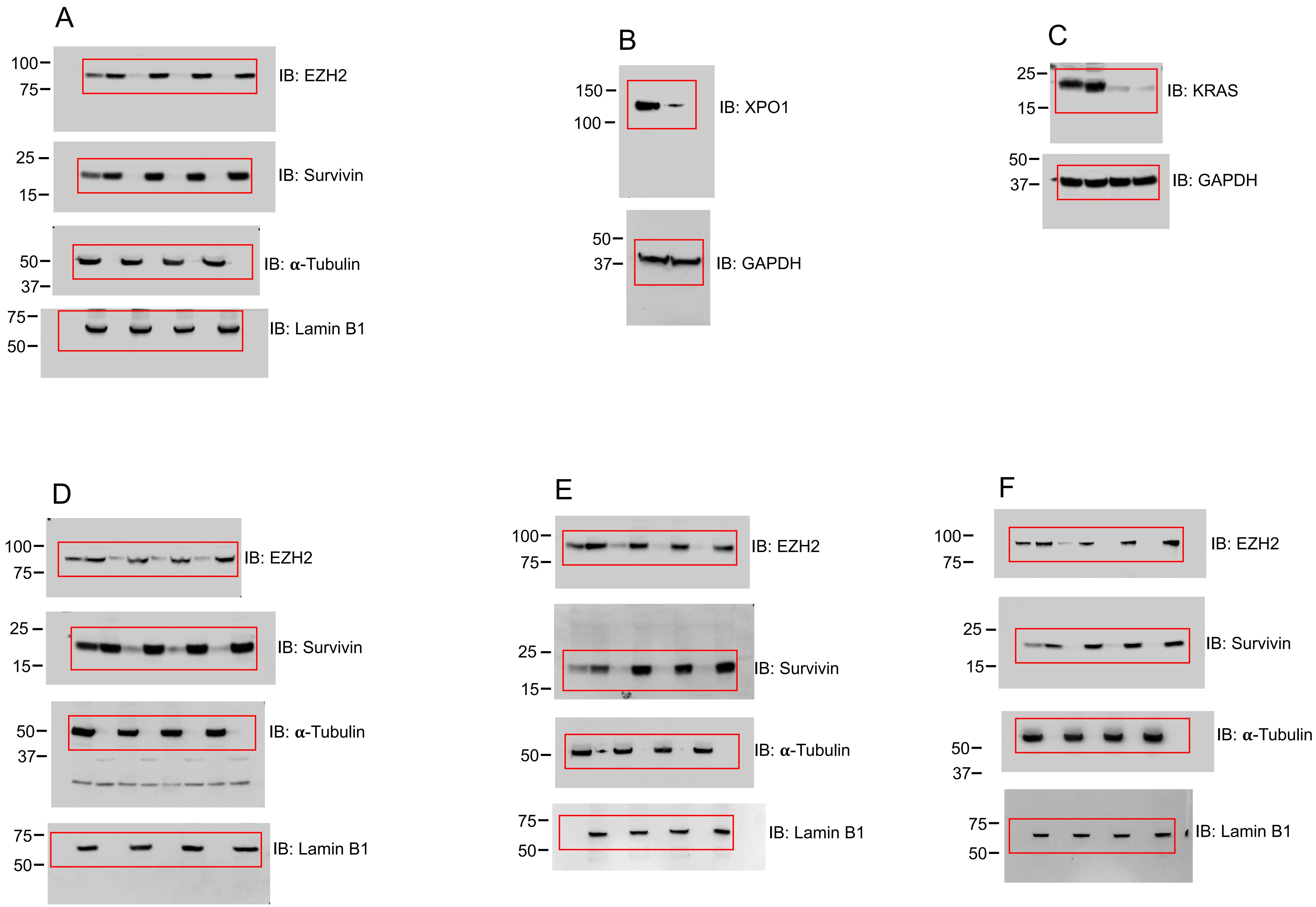

Uncropped blots for Extended Data Figure 3

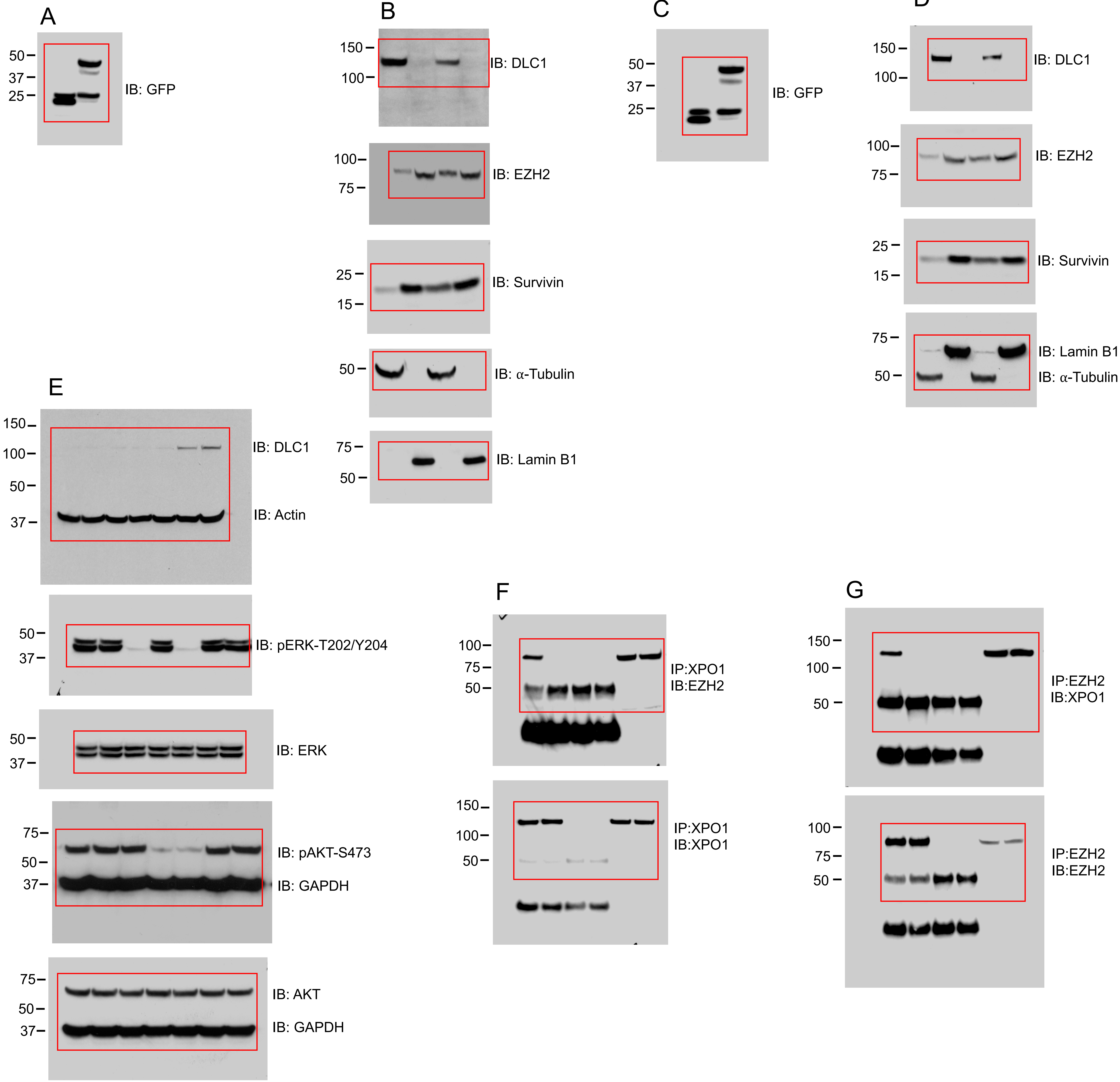

Uncropped blots for Extended Data Figure 3 continue

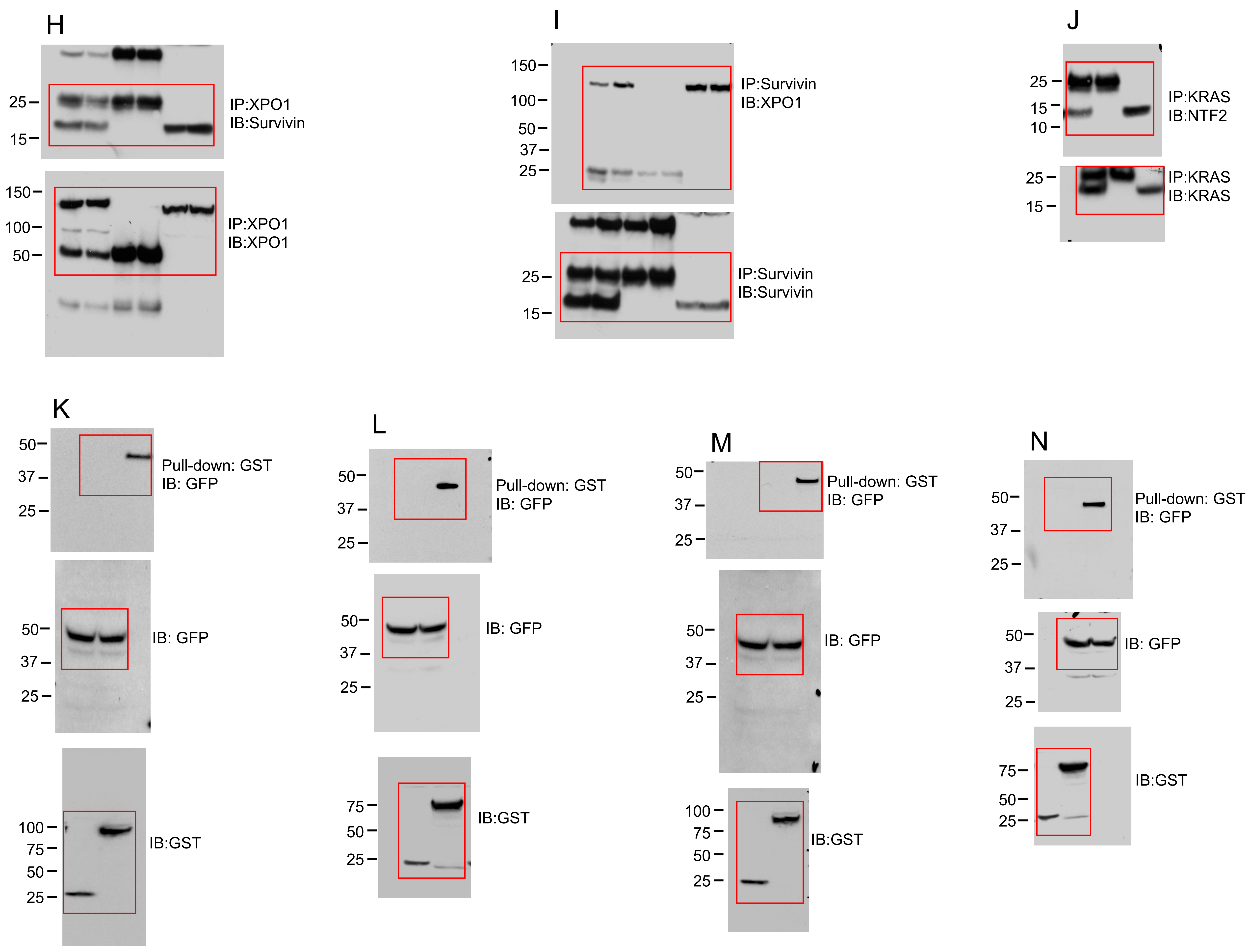

Uncropped blots for Extended Data Figure 6

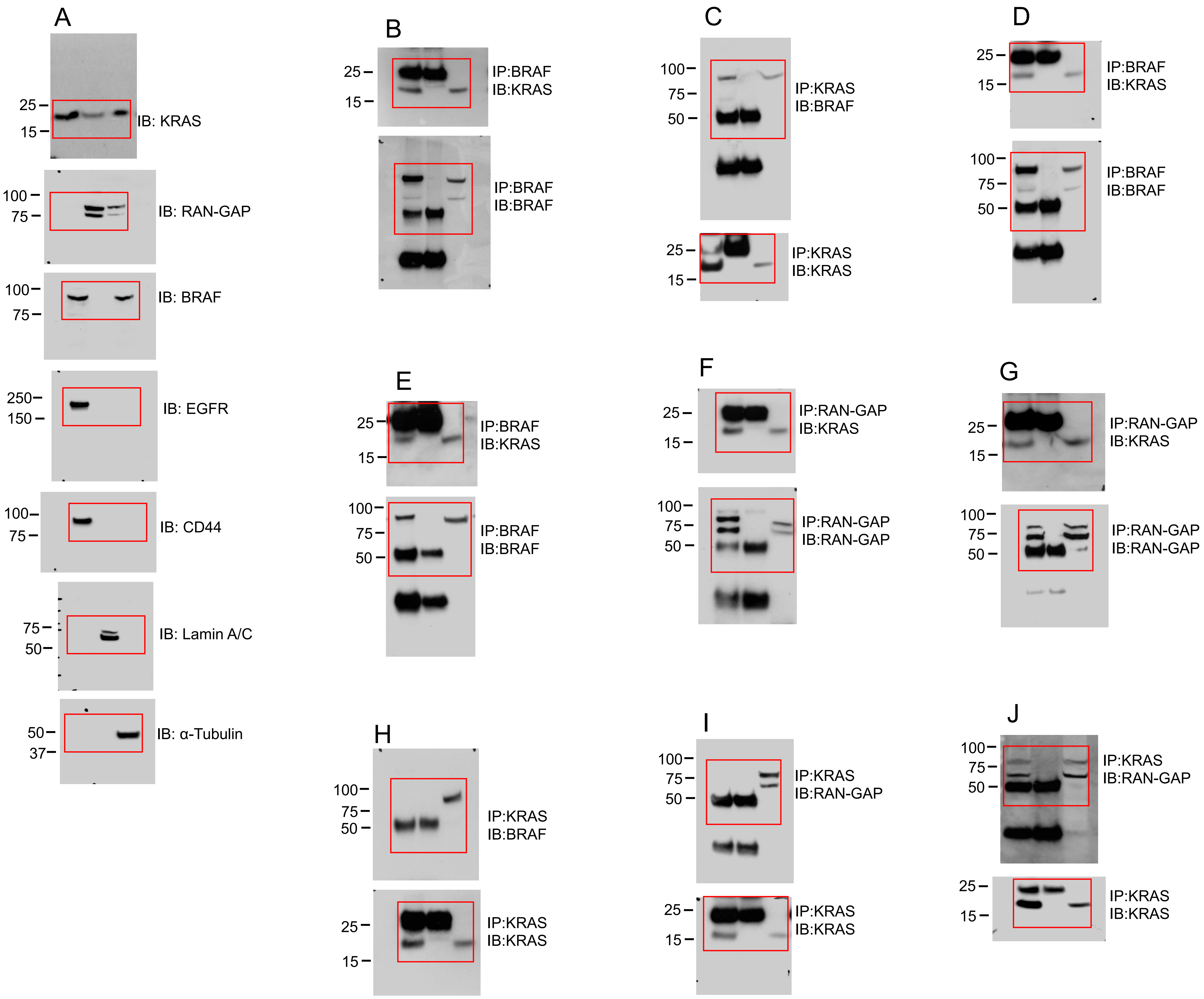

Uncropped blots for Extended Data Figure 6 continue

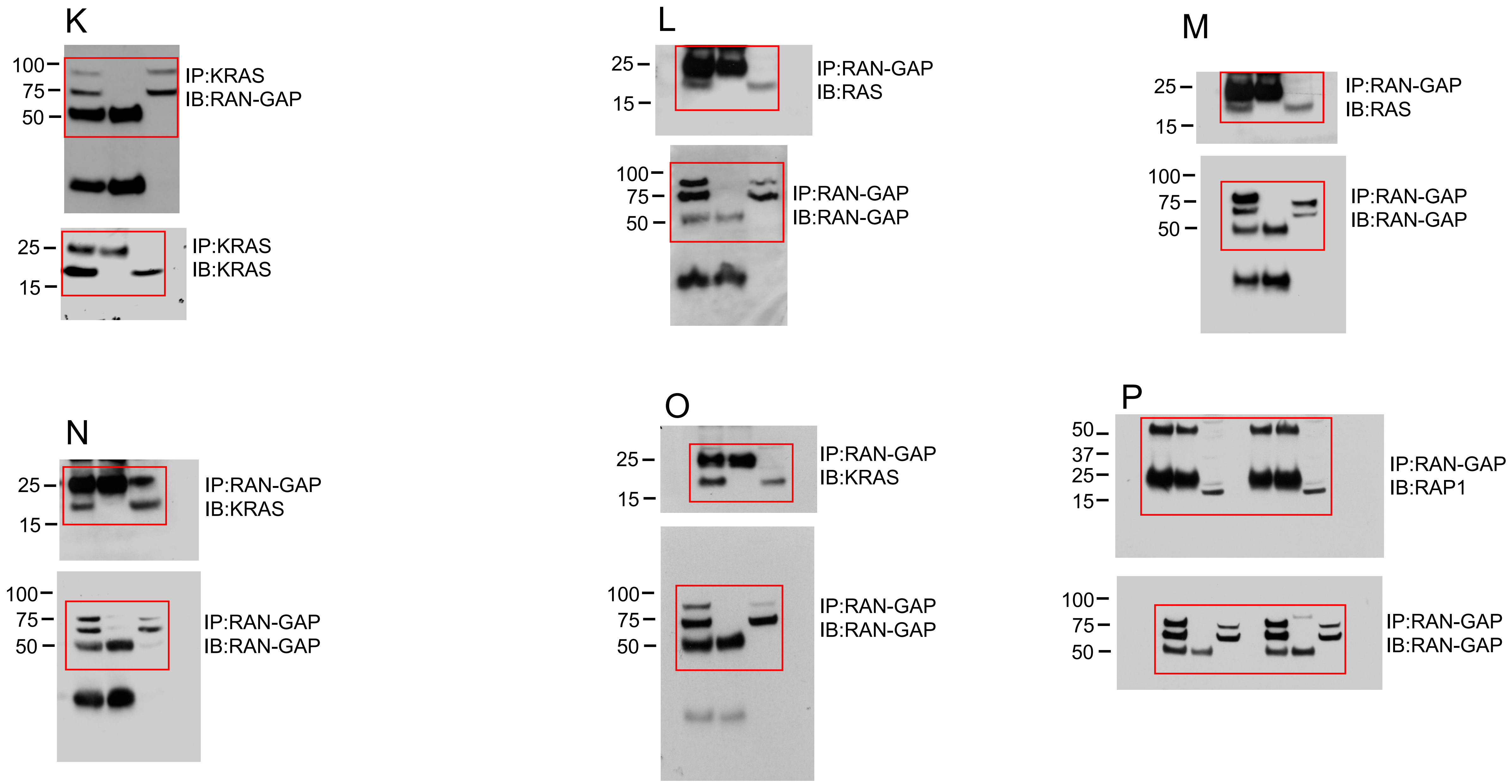

Supplement: Supplementary file 8 — Unprocessed and uncropped western blots and gels. [file 43018_2024_847_MOESM8_ESM.pdf]
